# The Phosphoinositide 3-Kinase Pathway in Human Cancer: Genetic Alterations and Therapeutic Implications

**DOI:** 10.2174/138920207782446160

**Published:** 2007-08

**Authors:** Alexandre Arcaro, Ana S Guerreiro

**Affiliations:** Division of Clinical Chemistry and Biochemistry, University Children’s Hospital Zurich, Steinwiesstrasse 75, CH-8032 Zurich, Switzerland

## Abstract

The phosphoinositide 3-kinase (PI3K) pathway is frequently activated in human cancer and represents an attractive target for therapies based on small molecule inhibitors. PI3K isoforms play an essential role in the signal transduction events activated by cell surface receptors including receptor tyrosine kinases (RTKs) and G-protein-coupled receptors (GPCRs). There are eight known PI3K isoforms in humans, which have been subdivided into three classes (I-III). Therefore PI3Ks show considerable diversity and it remains unclear which kinases in this family should be targeted in cancer. The class I_A_ of PI3K comprises the p110α, p110β and p110δ isoforms, which associate with activated RTKs. In human cancer, recent reports have described activating mutations in the *PIK3CA *gene encoding p110α, and inactivating mutations in the phosphatase and tensin homologue (*PTEN)* gene, a tumour suppressor and antagonist of the PI3K pathway. The *PIK3CA* mutations described in cancer constitutively activate p110α and, when expressed in cells drive oncogenic transformation. Moreover, these mutations cause the constitutive activation of downstream signaling molecules such as Akt/protein kinase B (PKB), mammalian target of rapamycin (mTOR) and ribosomal protein S6 kinase (S6K) that is commonly observed in cancer cells. In addition to p110α, the other isoforms of the PI3K family may also play a role in human cancer, although their individual functions remain to be precisely identified. In this review we will discuss the evidence implicating individual PI3K isoforms in human cancer and their potential as drug targets in this context.

## INTRODUCTION

Phosphoinositide 3-kinases (PI3Ks) are a family of signaling enzymes which regulate a variety of important cellular functions, including growth, cell cycle progression, apoptosis, migration, metabolism and vesicular trafficking [1, 2]. Since human cancer cells often display abnormal regulation of these cellular processes, the realization that PI3K signaling is disrupted at multiple levels has prompted researchers to develop targeted therapies against individual enzymes involved in this signaling cascade [3-6]. In this review, we will first discuss the PI3K signaling pathway and its functions in apoptosis, growth, cell cycle, angiogenesis, invasion and autophagy. We will subsequently present the main lines of evidence implicating genetic alterations in the PI3K signaling cascade in the development of human cancer and discuss some of the strategies that have been used to develop new cancer therapies based on targeting PI3K isoforms.

## PI3K ACTIVATION BY RECEPTOR TYROSINE KINASES

Phosphoinositide 3-kinase (PI3K) was first described 20 years ago as a distinct enzymatic activity associating with activated receptor tyrosine kinases (RTKs), such as the platelet-derived growth factor receptor (PDGFR) or with the polyoma virus middle T protein/pp60(c-src)complex [7-10].

PI3K activity was found to be elevated after cellular transformation by p60(v-src) [11] or abl [12]. After biochemical purification [13], the fist genes encoding the bovine catalytic p110α and regulatory p85α/β subunits of PI3K were cloned [14-17]. PI3K was shown to bind to activated RTKs *via* interaction of the Src homology-2 (SH2) domains of the p85 subunit to specific phosphotyrosine residues in the cytoplasmic domains of RTKs [15-22]. PI3K was then shown to be recruited to a broad variety of activated RTKs, including c-Met [23-25], c-Kit [26, 27], insulin-like growth factor-I receptor (IGF-IR) [28-30], insulin receptor (IR)/insulin receptor substrate-1 (IRS-1) [31-34], HER2/Neu/ErbB-2 [35], ErbB-3 [36-38], PDGFR [39, 40], Trk [41-43], and Flt3 [44]. 

Constitutively activated RTKs were found to be associated with PI3K, such as for c-Kit in leukemia [45], Tpr-Met [46] and EGFRvIII [47]. The constitutively activated BCR-ABL tyrosine kinase fusion protein which has been shown to be an essential step in the pathogenesis of Philadelphia chromosome (Ph)-positive leukemias also associates with PI3K [48]. In addition, PI3K interacts with Ras and is directly activated by Ras binding to p110 [49-51]. PI3K activation by RTKs such as the PDGFR was also reported to be regulated by Ras [52]. It was also shown that p85 contains a GTPase-responsive domain and an inhibitory domain, which together form a molecular switch that regulates PI3K [53]. H-Ras and Rac1 activate PI3K by targeting the GTPase-responsive domain [53]. The stimulatory effect of these molecules, however, is blocked by the inhibitory domain, which functions by binding to tyrosine-phosphorylated molecules and is neutralized by tyrosine phosphorylation [53]. The complementary effects of tyrosine kinases and small GTPases on the p85 molecular switch result in synergy between these two classes of molecules toward the activation of the PI3K/Akt pathway [53]. Another study showed that p85 inhibits p110 activation by Ras [54]. This blockage was released by Tyr kinase stimulation, showing that the classical mechanism of class I_A_ PI3K stimulation mediated by Tyr kinases also regulates Ras-induced PI3K activation [54]. At the same time as the genes of PI3K were cloned, it was shown that stimulation of cells with polypeptide growth factors such as PDGF induced the synthesis of novel second messengers phosphatidylinositol-3,4,5-trisphosphate (PIP_3_) and phosphatidylinositol-3,4-bisphosphate (PI(3,4)P_2_) [55, 56], which were resistant to the action of phospholipase C (PLC) [57]. 

## PI3K ISOFORMS

A family of PI3K isoforms was subsequently cloned and characterized [58-60]. These enzymes are subdived in three classes (I-III), based on sequence homology and *in vitro* substrate specificity. The class I_A_ of PI3K includes p85α/β and distinct regulatory subunits including mouse p55^PIK^ [61], and splice variants of the p85α gene such as p55α [62], p50α [63], human p55γ [64]. Moreover, in addition to p110α distinct catalytic p110 isoforms were cloned and termed p110β [65] and p110δ [66, 67].

G-protein-coupled receptors (GPCRs) were shown to activate the generation of PIP_3_ [68, 69], through the activation of a distinct isoform of PI3K. This distinct class I_B_ p110 isoform is activated by Gβγ subunits [70] and was termed p110γ [71, 72]. The p110γ isoform associates with an adaptor molecule (p101) contributing to its regulation by Gβγ subunits [72]. It should be also noted that the p110β isoform of class I_A_ PI3K has also been reported to be regulated by Gβγ [73]. Ras activates p110γ at the level of the membrane, by allosteric modulation and/or reorientation of the p110γ, implying that Ras can activate p110γ without its membrane translocation [74]. This view is supported by structural work that has suggested binding of Ras to p110γ results in a change in the structure of the catalytic pocket [75].

A separate class II of PI3Ks was identified in *Drosophila* and mammalian cells, which is characterized by a C-terminal C2 domain and a substrate specificity restricted to PI and PI(4)P *in vitro* [76-80]. This family includes the human PI3KC2α, PI3KC2β and PI3KC2γ isoforms [81-84].

These class III PI3Ks are homologues of the yeast VPS34 gene product (Vps34p) [85], which forms a complex with the Vps15 protein kinase and is essential for protein sorting to the yeast lysosome-like vacuole [86]. The human homologues of Vps34p and Vps15p were subsequently cloned [87, 88], as well as their *Drosophila* counterpart [89]. The human Vps34p was reported to associate with the trans-Golgi network, a key site for the formation of transport vesicles destined for different intracellular compartments [90]. 

A family of protein kinases with homology to PI3Ks was also cloned and characterized, including yeast target of rapamycin (TOR) proteins [91, 92], and their mammalian homologue FRAP/RAFT1/mTOR [93-96]. This family of protein kinases also includes ATM, the gene product that is mutated in the autosomal recessive disorder ataxia telangiectasia (AT) [97, 98], ATR, and DNA-dependent protein kinase (DNA-PK) [99].

## STRUCTURE OF PI3K ISOFORMS

The crystal structure of the SH2 and SH3 domains of p85α was solved, providing the first insights into the molecular mechanisms of PI3K regulation by RTKs [100, 101]. The interaction between the p85 and p110 subunits was shown to involve the inter-SH2 region of p85 [102, 103] and the N-terminal region of p110 [104, 105]. Furthermore, the p85 subunit contains two proline-rich sequences in its N-terminal region which can bind SH3 domains, such as those present in Src family kinases [106, 107]. Subsequently, the structure of the p85α breakpoint cluster region (BCR)-homology domain was solved [108]. The BCR domain is responsible for the activity of GTPase-activating proteins (GAP) in proteins such as BCR. The X-ray crystallographic structure of the catalytic isoform p110γ was then solved [109]. p110γ has a modular organization centred around a helical-domain spine, with C2 and catalytic domains positioned to interact with phospholipid membranes, and a Ras-binding domain placed against the catalytic domain where it could drive allosteric activation of the enzyme [109]. A crystal structure of a p110γ/Ras complex was also reported [75]. A critical loop in the Ras binding domain positions Ras so that it uses its switch I and switch II regions to bind p110γ [75]. Ras also forms a direct contact with the p110γ catalytic domain [75]. The complex with Ras shows a change in the PI3K conformation that may represent an allosteric component of Ras activation. The X-ray crystallographic structures of p110γ bound to the specific PI3K inhibitors wortmannin and LY294002 and to the broad-spectrum protein kinase inhibitors quercetin, myricetin, and staurosporine reveal how these compounds fit into the ATP binding pocket. With a nanomolar IC_50_, wortmannin most closely fits and fills the active site and induces a conformational change in the catalytic domain [110]. These results provided the basis for development of isoform-specific PI3K inhibitors with therapeutic potential [110].

## PI3K SIGNALING

The use of mutant receptors defined for the first time a role for PI3K in PDGF-dependent DNA synthesis, and established PI3K as an independent downstream mediators of PDGF's mitogenic signal [111]. Furthermore, a mutant CSF-1R with a mutation in the PI3K-binding site had impaired ability to transduce signals controlling changes in morphology and increased cell growth [112]. The PI3K binding sites also appeared both necessary and sufficient for the normal endocytic trafficking of the activated PDGFR [113]. A PDGFR mutant in which both p85-binding sites were mutated failed to stimulate membrane ruffling and chemotaxis, suggesting a role for PI3K in these responses [114, 115]. 

**Fig. (1).**
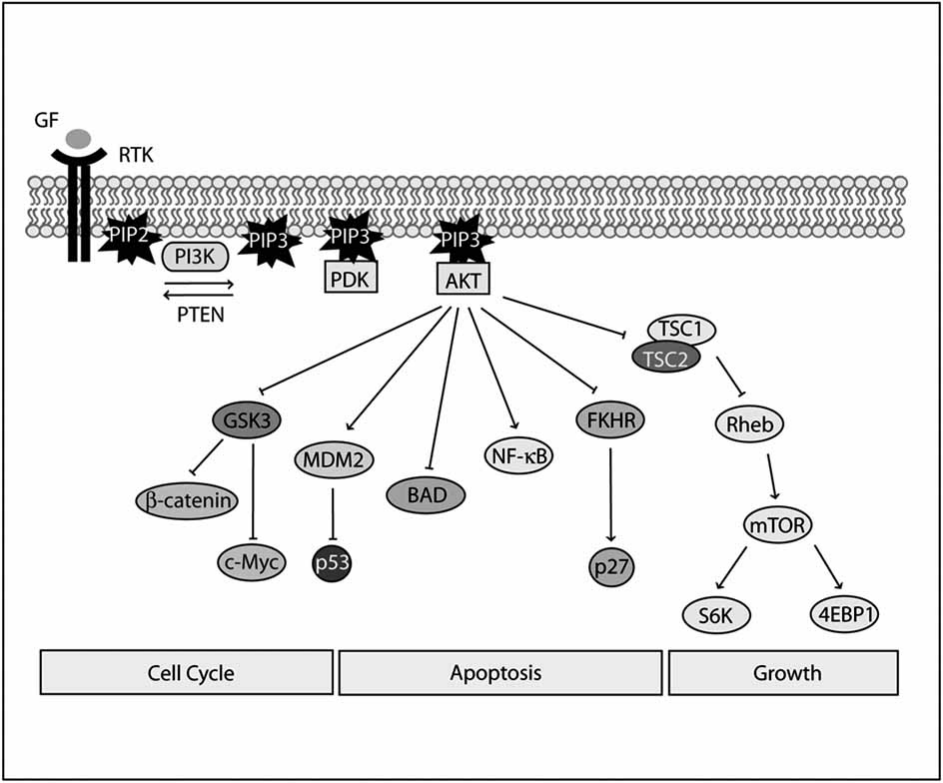
**Signalling through the phosphatidylinositol 3-kinase (PI3K) affects cell growth, apoptosis and cell cycle regulation.** The PI3K/Akt related pathways have a key role in initiating intracellular signalling cascades subsequent to the activation of membrane tyrosine kinases. The PI3K phosphorylates phosphatidylinositol-biphosphates (PIP_2_), generating phosphatidylinositol-triphosphates (PIP_3_). PIP_3_ act as docking sites for Akt and PDK at the plasma membrane. Upon phosphorylation by PDK, AKT becomes activated and phosphorylates in turn several downstream proteins, regulating cell growth, survival, apoptosis and cell cycle. PTEN, phosphatase and tensin homolog deleted on chromosome 10; PDK, phosphoinositide-dependent kinases; GSK3, glycogen synthase kinase-3; MDM2, murine double minute; FKHR, forkhead; NF-κB, nuclear factor κB; Rheb, Ras homologue enriched in the brain; TSC1, TSC2, tuberous sclerosis complex 1 and 2; mTOR, mammalian target of rapamycin; 4EBP1, eukaryotic translation initiation factor 4E binding protein; S6K, S6 kinase.

Pharmacological inhibitors of PI3K were instrumental in elucidating the role of the enzyme in cellular signaling events. These inhibitors include quercetin analogs, the most widely used being LY294002 [116], as well as the microbial product wortmannin [117-119]. In addition to their effects on PI3K isoforms, LY294002 and wortmannin also inhibit the activity of the related kinases such as mTOR [120], ATM and DNA-PK [121]. Studies with wortmannin and LY294002 uncovered a role for PI3K in activation of p70(S6K) by insulin and PDGF [122-125], as well as in the inactivation of glycogen synthase kinase-3 (GSK-3) [126]. Subsequently, PI3K was shown to be essential in the activation of the proto-oncogene Akt (or protein kinase B (PKB)) by PDGF [127] and other growth factors [128, 129]. These results were confirmed by the observation that transfection of membrane-targeted p110 was sufficient to trigger downstream responses characteristic of growth factor action, including the stimulation of p70(S6K) and Akt [130, 131]. The phospholipids products of PI3K, initially PI(3,4)P_2_, were shown to directly activate Akt by binding to its pleckstrin homology (PH) domain [132, 133]. PH domains were recognized to be modular domains with the ability to specifically bind to the lipid products of PI3K, including PIP_3_ [134]. PI3K activity was also required for phosphorylation of both Thr308 and Ser473 activation sites of Akt [135]. The kinase that phosphorylates Akt was then purified, cloned and shown to phosphorylate Akt1 at Thr308 and increase its activity [136-138]. It was found that only PI(3,4,5)P_3_ or PI(3,4)P_2_ were effective in potently activating the kinase, which was termed PI(3,4,5)P_3_-dependent protein kinase-1 (PDK1) [136]. PDK1 is the protein kinase that mediates the activation of Akt/PKB by insulin and growth factors [139]. PDK1 therefore plays a key role in mediating many of the actions of the second messengers produced by PI3K. In response to PDGF, binding of PI(3,4,5)P_3_ and/or PI(3,4)P_2_ to the PH domain of PDK-1 causes its translocation to the plasma membrane where it co-localises with Akt/PKB, significantly contributing to the scale of Akt/PKB activation [139, 140] (Fig. **1**). The identification of the kinase that phosphorylates the Akt Ser473 was achieved only in the last few years [141]. The complex of the mammalian target of rapamycin (mTOR) and Rictor was shown to be essential for this crucial phosphorylation step in Akt by several groups [141, 142]. 

In addition to its role in Akt activation, PDK1 was also shown to be responsible for the regulation of other protein kinases [143, 144]. PDK1 phosphorylated the activation loop sites of PKCξ and PKCδ *in vitro* and in a PI3K-dependent manner *in vivo* [145, 146]. Several members of the PKC family tested formed complexes with PDK1 [145]. Serum and glucocorticoid-inducible kinase (SGK) was also shown to be a target of PI3K/PDK1 [147, 148]. A regulatory link between p70(S6K) and PDK1 was also described, since PDK1 selectively phosphorylates and activated p70(S6K)* in vitro* and *in vivo *[149, 150]. 

In addition, PI3K was reported to be involved in the activation of several other protein kinases, including c-Jun N-terminal kinase (JNK) by EGF [151]. Bruton's tyrosine kinase (Btk), which has a PH domain that can bind PIP_3_ [152], was described as a downstream target of PI3K (p110γ) [153]. Etk/Bmx a member of the Btk tyrosine kinase family that contains a PH domain is also involved in the PI3K pathway [154]. The Tec family non-receptor tyrosine kinases were shown to be regulated by PIP_3_ interacting with its PH domain [155]. Activation of PI3K caused phospholipase C-γ (PLC-γ) PH domain-mediated membrane targeting and PLC-γ activation [156, 157]. Integrin-linked kinase (ILK) was also proposed to be a receptor-proximal effector for the PI3K-dependent, extracellular matrix and growth factor mediated, activation of Akt, and inhibition of GSK-3 [158]. RNA interference (RNAi) as well as conditional knock-out of ILK had no effect on phosphorylation of Akt on Thr-308 but resulted in almost complete inhibition of phosphorylation on Ser-473 and significant inhibition of Akt activity, accompanied by significant stimulation of apoptosis [159]. In addition, Raf-1 activation by Ras was shown to be achieved through a combination of both physical interaction and indirect mechanisms involving the activation of PI3K as a second Ras effector, which directs p21-activated kinase (PAK)-mediated regulatory phosphorylation of Raf-1 [160]. Phosphorylation of Raf-1 on Ser338 through PI3K and Pak was also shown to provide a co-stimulatory signal which together with Ras leads to strong activation of Raf-1 kinase activity by integrins [161].

A consensus sequence which predicts high-affinity binding of PH domains to PtdIns(3, 4)P_2_ and/or PtdIns(3,4,5)P_3_ was proposed, and several new PH domain-containing proteins that directly bind PI3K products were identified, including Gab1, Dos, myosinX, and Sbf1 [162], GAP1(m) a member of the GAP1 family of Ras GTPase-activating proteins (GAPs) [163], DAPP1 [164], Tec family tyrosine kinases [165], ARAP3 [166], and P-Rex1, a Rac activator [167]. 

## PI3K /Akt SIGNALING AND APOPTOSIS

The involvement of PI3K in prevention of apoptosis by polypeptide growth factor receptors was first described by studies using both wortmannin and LY294002 [168-170]. Experiments with pharmacological inhibitors, as well as expression of wild-type and dominant-inhibitory forms of Akt, demonstrated that Akt mediates PI3K-dependent survival [171-175]. These findings were supported by studies showing that Ras activation of PI3K suppresses c-Myc-induced apoptosis through the activation of Akt but not p70(S6K) [176]. UV-B light-induced-apoptosis was also prevented by IGF-I/PI3K/Akt signaling [177] and interleukin-3-dependent survival of hematopoietic cells required PI3K/Akt signaling [178]. Neuronal survival in the absence of nerve growth factor (NGF) was promoted by PI3K/Akt [179] and it was also shown that Akt can transduce a survival signal for differentiating neuronal cells through a mechanism that is independent of induction of Bcl-2 or Bcl-X_L_, or inhibition of JNK activity [180]. PI3K acting through Akt was implicated as a key mediator of the aberrant survival of Ras-transformed epithelial cells in the absence of attachment, and as a mediator of matrix-induced survival of normal epithelial cells [181]. 

Some of the proposed mechanisms for the antiapoptotic effect of activated Akt include the inhibition of proapoptotic Bcl-2 family proteins, downregulation of death receptors, and enhancement of the glycolytic rate [182]. There exists a large panel of Akt substrates which mediate its effects on cellular responses, including apoptosis, growth and cell cycle regulation [173-175] (Fig. **1**). The Akt targets identified so far include BAD [183-185], the FOXO (Forkhead Box, subgroup O) family of transcription factors [186-189] and AFX [190], glycogen synthase kinase-3 (GSK-3) [139, 191, 192], p27(Kip1) [193, 194], Mdm2 [195], endothelial NO synthase (eNOS) [196, 197], cyclic nucleotide phosphodiesterase 3B isoform (PDE3B)[198], Raf [199, 200], apoptosis signal-regulating kinase 1 (ASK1) [201], androgen receptor (AR) [202], the nuclear factor CREB [203], the p300 transcriptional coactivator [204] and E2F [205, 206]. 

In addition, it was shown that Akt can regulate signaling pathways that lead to induction of the NF-κB family of transcription factors [207-209]. This induction occurred at the level of degradation of the NF-κB inhibitor IκB [207]. PDGF was also shown to activate NF-κB through Ras and PI3K to Akt and the IκB kinase (IKK) [208]. Upon PDGF stimulation, Akt transiently associated *in vivo* with IKK and induced IKK activation [208]. Akt was reported to stimulate NF-κB predominantly by up-regulating of the transactivation potential of the p65 subunit of NF-κB [210]. Survivin, a member of the inhibitors-of-apoptosis gene family, is expressed in a cell-cycle-dependent manner in all the most common cancers but not in normal differentiated adult tissues [211]. Hematopoietic cytokines were reported to exert their antiapoptotic and mitogenic effects, at least in part, by increasing survivin levels, which was dependent on PI3K [211]. It was also shown that both vascular endothelial growth factor (VEGF) and basic fibroblast growth factor significantly reduce the pro-apoptotic potency of chemotherapy on endothelial cells, a response, which PI3K-dependent and could be recapitulated by over-expressing the dominant-active form of Akt [212]. Work by others showed that the anti-apoptotic effects of IL-6 were mediated, at least in part, by Mcl-1 (Bcl-2 family member) expression and that the response occured mainly through the PI3K/Akt pathway [213]. It was reported that apoptotic cell death of PTEN-deficient prostate cancer cells induced by LY294002 or expression of wild type PTEN can be abrogated by disrupting Fas/Fas ligand (FasL) interactions [214]. These data showed that apoptosis induced by blockade of the PI3K pathway in prostate tumor cells is mediated by an autocrine Fas/FasL apoptotic mechanism and that the Fas apoptotic pathway is both necessary and sufficient to mediate apoptosis by PI3K inhibition [214].

## PI3K SIGNALING AND GROWTH CONTROL

Signaling networks that promote cell growth are frequently dysregulated in cancer. One regulatory network, which converges on effectors such as eIF4E-binding proteins-1 (4E-BP1) and p70(S6K), leads to growth by promoting protein synthesis [215]. In particular, a tumor suppressor complex whose function is lost in tuberous sclerosis patients regulates the nutrient signal carried by the critical signaling protein TOR to the effectors 4E-BP1 and p70(S6K) [215]. 

It was initially demonstrated that the PI3K/Akt signaling pathway, in concert with FRAP/mTOR, induces the phosphorylation and inactivation of the translational repressor, the 4E-BP1 [216] and activation of p70(S6K) [217, 218]. Further work showed that mTOR signals downstream to at least two independent targets, S6K1 and 4E-BP1/eIF4E that function in translational control to regulate mammalian cell size [219]. The tuberous sclerosis complex-2 (TSC2) gene product, tuberin, is as a target of Akt [220-223]. Normal cellular functions of hamartin and tuberin, encoded by the TSC1 and TSC2 tumor suppressor genes, are closely related to their direct interactions. Tuberous sclerosis (TSC) is an autosomal dominant disorder characterized by the formation of hamartomas in a wide range of human tissues [224]. It was demonstrated that, upon activation of PI3K, tuberin is phosphorylated on consensus recognition sites for PI3K-dependent S/T kinases [220]. Moreover, Akt/PKB could phosphorylate tuberin *in vitro* and *in vivo* [220]. It was also shown that S939 and T1462 of tuberin are PI3K-regulated phosphorylation sites and that T1462 is constitutively phosphorylated in *PTEN* (-/-) tumor-derived cell lines [220]. Finally, a tuberin mutant lacking the major PI3K-dependent phosphorylation sites blocked the activation of S6K1, suggesting a means by which the PI3K-Akt pathway regulates S6K1 activity [220]. Other reports showed that TSC1-TSC2 inhibits the p70(S6K) and activates the 4E-BP1, which was mediated by inhibition of mTOR [220, 222]. Furthermore, Tsc2 was shown to be directly phosphorylated by Akt. Tsc2 was inactivated by Akt-dependent phosphorylation, which destabilizes Tsc2 and disrupts its interaction with Tsc1 [222]. It was shown that TSC1 and TSC2 antagonize the amino acid-TOR signaling pathway [221]. Tsc1 and Tsc2 could physically associate with TOR and function upstream of TOR genetically. In *Drosophila melanogaster* and mammalian cells, loss of *TSC1* and *TSC2* resulted in a TOR-dependent increase of S6K activity [225]. Furthermore, although S6K is normally inactivated in animal cells in response to amino acid starvation, loss of *TSC1*-*TSC2* renders cells resistant to amino acid starvation. It was thus proposed that the Tsc1-Tsc2 complex antagonizes the TOR-mediated response to amino acid availability [225]. These studies identified Tsc1 and Tsc2 as regulators of the amino acid-TOR pathway and provide a new paradigm for how proteins involved in nutrient sensing function as tumor suppressors [221, 225]. Work by another group showed that insulin or IGF-I stimulated phosphorylation of tuberin, which was inhibited by the PI3K inhibitor LY294002 [226]. Expression of constitutively active PI3K or active Akt induced tuberin phosphorylation. It was further demonstrated that Akt/PKB associates with hamartin-tuberin complexes, promoting phosphorylation of tuberin and increased degradation of hamartin-tuberin complexes [226]. The ability to form complexes, however, was not blocked. Akt also inhibited tuberin-mediated degradation of p27(KIP1), thereby promoting CDK2 activity and cellular proliferation [226]. These results confirmed that tuberin is a direct physiological substrate of Akt and that phosphorylation of tuberin by PI3K/Akt is a major mechanism controlling hamartin-tuberin function [226].

Further work showed that TSC1/2 is a GAP for the small GTPase Rheb and that insulin-mediated Rheb activation is PI3K-dependent [227-229]. Rheb over-expression induced S6K1 phosphorylation and inhibited Akt phosphorylation, as did loss-of-function mutations in TSC1/2 [227]. Co-expression of a human TSC2 harboring a disease-associated point mutation in the GAP domain, failed to stimulate Rheb GTPase activity or block Rheb activation of S6K1 [227, 229]. A screen for novel regulators of growth identified Rheb (Ras homologue enriched in brain), a member of the Ras superfamily of GTP-binding proteins [228]. Increased levels of Rheb in *Drosophila melanogaster* promoted cell growth and alter cell cycle kinetics in multiple tissues. In mitotic tissues, overexpression of Rheb accelerates passage through G1-S phase without affecting rates of cell division [228]. Genetic and biochemical tests indicated that Rheb functions in the insulin signalling pathway downstream of Tsc1-Tsc2 and upstream of TOR [228]. In another study, mutations in the *Drosophila melanogaster Rheb* gene were isolated as growth-inhibitors, whereas over-expression of Rheb promoted cell growth [230]. Genetic and biochemical analyses suggest that Rheb functions downstream of the tumour suppressors Tsc1-Tsc2 in the TOR signalling pathway to control growth, and that a major effector of Rheb function is S6K [230, 231].

It was reported that Akt activation causes proteasomal degradation of substrates that control cell growth and survival [232]. Expression of activated Akt triggered proteasome-dependent declines in the protein levels of the Akt substrates tuberin, FOXO1, and FOXO3a [232]. The addition of proteasome inhibitors stabilized the phosphorylated forms of multiple Akt substrates, including tuberin and FOXO proteins [232]. Activation of Akt also triggered the ubiquitination of several proteins containing phosphorylated Akt substrate motifs [232]. Together the data indicate that activated Akt stimulates proteasomal degradation of its substrates and suggest that Akt-dependent cell growth and survival are induced through the degradation of negative regulators of these processes [232]. It has been shown that FKHR is phosphorylated *via* insulin or growth factor signaling cascades, resulting in its cytoplasmic retention and the repression of target gene expression. Insulin treatment was shown to decrease endogenous FKHR proteins in HepG2 cells, which was inhibited by proteasome inhibitors [233]. FKHR was ubiquitinated *in vivo* and *in vitro*, and insulin enhances the ubiquitination in the cells [233]. In addition, the signal to FKHR degradation from insulin was shown to be mediated by the PI3K pathway, and the mutation of FKHR at the serine or threonine residues phosphorylated by Akt inhibited the ubiquitination *in vivo* and *in vitro* [233]. Another report showed that FoxO1 expression is constitutively suppressed in chicken embryo fibroblasts transformed by P3k or Akt [234]. In this system, phosphorylation-dependent degradation of FoxO1 by means of proteasomes played a role in oncogenic transformation by P3k and Akt [234].

It was shown that Tsc1-Tsc2 is required for insulin signaling to PI3K [235, 236] by restraining the activity of S6K1, which when activated inactivates insulin receptor substrate (IRS) function, *via* repression of IRS-1 gene expression and *via* direct phosphorylation of IRS-1 [235]. These results suggested that the low malignant potential of tumors arising from TSC1-2 dysfunction may be explained by the failure of TSC mutant cells to activate PI3K and its downstream effectors [235, 236].

## PI3K IN CELL CYCLE CONTROL

Cell cycle progression is a tightly controlled process. To initiate cell division, mitogens trigger a number of early signals that promote the G(0)-G(1) transition by inducing cell growth and the activation of G(1) cyclins. Activation of cyclin E/cdk2 (cyclin-dependent kinase 2) at the end of G(1) is then required to trigger DNA synthesis (S phase entry). Among the early signals induced by mitogens, activation of PI3K appears essential to induce cell cycle entry, as it regulates cell growth signalling pathways (see previous section), which in turn determine the rate of cell cycle progression. Another mechanisms by which PI3K and its downstream effector Akt regulate cell cycle entry is by inactivation of the FOXO transcription factors, which induce expression of quiescence genes such as those encoding p27(Kip), p130 and cyclin G2 [237]. PI3K/FOXO then work as a complementary switch: when PI3K is active, FOXO transcription factors are inactive [237]. The switch is turned on and off at different phases of the cell cycle, thus regulating cell cycle progression. Akt triggers a network that positively regulates G1/S cell cycle progression through inactivation of GSK3β, leading to increased cyclin D1, and inhibition of Forkhead family transcription factors and the tumor suppressor tuberin (TSC2), leading to reduction of p27(Kip1) [238]. The identification of p21Waf1/Cip1 and p27Kip1 as novel substrates of Akt provided new insights into mechanisms whereby hyperactivation of this lipid signaling pathway may lead to cell cycle deregulation in human cancers [238].

The PI3K/Akt pathway must be activated in G1 to inactivate forkhead transcription factors and allow cell cycle entry. It was subsequently shown that attenuation of the PI3K/Akt pathway is required to allow transcriptional activation of FOXO in G2 [239]. FOXO activity in G2 controls mammalian cell cycle termination, as interference with FOXO transcriptional activation by disrupting PI3K/Akt downregulation, or by expressing a transcriptionally inactive FOXO mutant, induces cell accumulation in G2/M, defective cytokinesis, and delayed transition from M to G1 of the cell cycle [239]. It was demonstrated that FOXO regulate expression of mitotic genes such as cyclin B and polo-like kinase (Plk) [239]. These results supported the important role of forkhead transcription factors in the control of mammalian cell cycle completion, and suggest that efficient execution of the mitotic programme depends on downregulation of PI3K/Akt and consequent induction of FOXO transcriptional activity [239].

## PI3K SIGNALING IN MIGRATION AND INVASION

Migration of cancer cells is one of the key factors responsible for cancer metastasis. The elucidation of mechanisms responsible for the highly invasive potential of cancer cells can help to identify specific targets for the treatment of cancer patients. Highly invasive cancers are usually characterized by aberrant activity of specific intra- or extracellular molecules such as protein kinases, phosphatases, transcriptional factors, proteolytic enzymes, and others. Therefore, inhibition of specific target molecules in common signaling pathway(s) responsible for metastatic spread can have potential clinical relevance. 

The first identified downstream target of PI3K in PDGF-stimulated membrane ruffling was Rac [240, 241]. Class I_A_ PI3Ks are implicated in many cellular responses controlled by receptor tyrosine kinases (RTKs), including actin cytoskeletal remodeling. Within this pathway, Rac is a key downstream target/effector of PI3K. One possible candidate for this function is the Rac-activating complex Eps8-Abi1-Sos-1, which possesses Rac-specific guanine nucleotide exchange factor (GEF) activity [242]. It was shown that Abi1 (also known as E3b1) recruits PI3K, *via* p85, into a multimolecular signaling complex that includes Eps8 and Sos-1 [242]. The recruitment of p85 to the Eps8-Abi1-Sos-1 complex and PIP_3_, co-operate to unmask its Rac-GEF activity *in vitro* [242]. Moreover, they are indispensable for the activation of Rac and Rac-dependent actin remodeling *in vivo* [242]. Upon growth factor stimulation, endogenous p85 and Abi1 consistently colocalized into membrane ruffles, and cells lacking p85 failed to support Abi1-dependent Rac activation [242].

Direct PI3K activation was sufficient to disrupt epithelial polarization and induce cell migration and invasion [243]. PI3K inhibition also disrupted actin structures, suggesting that activation of PI3K alters actin organization, leading to increased motility and invasiveness [243]. Integrin-mediated activation of PI3K was shown to promote carcinoma invasion by targeting Rac [244]. Vav, a guanosine diphosphate (GDP)-guanosine triphosphate (GTP) exchange factor (GEF) for Rac that stimulates the exchange of bound GDP for GTP, bound to and was directly controlled by substrates and products of PI3K [245]. PI3K also acts upstream of Tiam1, an activator of Rac [246]. Akt/PKB potently promoted invasion of highly metastatic cells, by increasing cell motility and matrix metalloproteinase-9 (MMP-9) production, in a manner highly dependent on its kinase activity and membrane-translocating ability [247]. The increase in MMP-9 production was mediated by activation of NF-κB transcriptional activity by Akt/PKB [247]. However, Akt/PKB did not affect the cell-cell or cell-matrix adhesion properties of the cells. These findings thus established Akt/PKB as a major factor in the invasive abilities of cancer cells [247]. Another study showed that PI3K is constitutively active and controls cell motility of highly invasive breast cancer cells by the activation of transcription factor, NF-κB [248]. The urokinase-type plasminogen activator (uPA) promoter contains an NF-κB binding site, and uPA expression in MDA-MB-231 cells was induced by the constitutively active NF-κB [248]. Cell migration was inhibited by overexpression of a dominant negative p85α, as well as by pretreatment of cells with wortmannin and LY294002 [248]. Highly invasive MDA-MB-231 cells constitutively secreted uPA in amounts significantly higher than poorly invasive MCF-7 cells [248].Furthermore, inhibition of NF-κB markedly attenuated endogenous migration, and inhibition of PI3K and NF-κB reduced secretion of uPA [248]. These data suggest a link between constitutively active PI3K, NF-κB, and secretion of uPA, which is responsible for the migration of highly invasive breast cancer cells [248]. In another study, constitutive activation of Akt was identified in breast cancer cells, while benign breast epithelial cell lines were immortalized through pathways that are independent of the EGF/PI3K/Akt kinase cascade, but this was not associated with invasiveness [249]. Transfection of constitutively active Akt caused accelerated cell division and osteopontin expression [249]. Conversely, dominant-negative Akt kinase slows cell cycle progression and suppresses osteopontin expression [249]. The manipulation of osteopontin expression in this setting by transfection of the gene or its antisense did not affect the growth rate of the cells but altered cell motility and anchorage independence [249]. Therefore, Akt kinase was postulated to activate two distinct genetic programs: the program of growth and survival and the program of invasiveness and anchorage independence, which is mediated by osteopontin [249]. These studies define Akt kinase as a molecular bridge between cell cycle progression and dissemination. In colorectal cancer, another group investigated the effect of inhibiting the PI3K/Akt/IKKα pathway in regulating the inappropriate constitutive activation of NF-κB and β-catenin [250]. Inducible expression of either dominant-negative IKKα or PTEN strongly inhibited both the constitutive NF-κB- and β-catenin-dependent promoter and endogenous gene activation [250]. Targeted array-based gene expression analysis of this inducible system reveals that many of the genes downregulated upon inhibition of this pathway were involved in tumor angiogenesis and metastasis [250].

## PI3K IN ANGIOGENESIS

PI3K signaling plays a key role in tumor angiogenesis, the development of new blood vessels. Hypoxia develops in the majority of solid tumors due to the inability of the existing vascular system to supply the growing tumor mass with adequate amounts of oxygen [251]. A large body of clinical evidence suggests that intratumoral hypoxia correlates with the elevated aggressive behavior of cancer cells and their resistance to therapy, leading to poor patient prognoses [251]. Hypoxia-inducible factor 1 (HIF-1) is a transcriptional activator that functions as a master regulator of O_2_ homeostasis [252]. HIF-1 target genes encode proteins that increase O_2_ delivery and mediate adaptive responses to O_2_ deprivation [252]. Several HIF-1 targets are known, including the gene encoding vascular endothelial growth factor (VEGF). HIF-1 activity is regulated by the cellular O_2_ concentration and by the major growth factor-stimulated signal transduction pathways [252]. In human cancer cells, both intratumoral hypoxia and genetic alterations affecting signal transduction pathways lead to increased HIF-1 activity, which promotes angiogenesis, metabolic adaptation, and other critical aspects of tumor progression [252, 253]. HIF-1 is a transcriptional activator composed of HIF-1α and HIF-1β subunits [253, 254]. Under hypoxic conditions, HIF-1α expression increases as a result of decreased ubiquitination and degradation [254]. The tumor suppressors VHL (von Hippel-Lindau protein) and p53 target HIF-1α for ubiquitination such that their inactivation in tumor cells increases the half-life of HIF-1α [254]. Increased PI3K and Akt or decreased PTEN activity in cancer cells were also shown to increase HIF-1α [255]. A further study observed that hypoxic induction of HIF-1α protein was decreased by serum deprivation in carcinoma cells under low oxygen tension [256]. Over-expression of dominant-active Akt1 restored HIF-1α expression, whereas inhibition of PI3K activity reduced hypoxic HIF-1α protein levels [256]. An immunohistochemical analysis of human breast cancers revealed that lack of Akt1 phosphorylation correlates with low HIF-1α levels [256]. The angiogenic switch in Ras-transformed cells is promoted by the tumor microenvironment through the induction of the angiogenic mitogen, VEGF. It was shown that hypoxia modulates VEGF induction in Ras-transformed cells through the activation of a stress inducible PI3K/Akt pathway and the HIF-1 transcriptional response element [257]. Hypoxia was reported to activate a growth factor receptor (PDGFR)/PI3K/Akt cascade that leads to GSK-3 inactivation, and thus impact on cell survival, proliferation, and metabolism [258]. It was also reported that under hypoxic conditions, rat pheochromocytoma PC12 cells are resistant to apoptosis induced by serum withdrawal and chemotherapy treatment. The hypoxia-dependent protection from apoptosis correlated with activation of the PI3K/Akt pathway [259]. In another study, insulin and epidermal growth factor or an inactivating mutation in the tumor suppressor *PTEN* specifically increased the protein levels of HIF-1α, but not of HIF-1β in human cancer cell lines [260]. This specific elevation of HIF-1α protein expression required PI3K signaling [260]. Another study examined the involvement of FOXO4 (also known as AFX), a member of the forkhead transcription factor superfamily that is negatively regulated by the PI3K/Akt pathway, in the regulation of HIF-1α protein expression [261]. Nuclear expression of FOXO4 resulted in the suppression of various responses to hypoxia, including decreased VEGF [261]. Interestingly, FOXO4 down-regulated the HIF-1α protein levels, consistent with the lack of hypoxia responsiveness [261]. In another study, the linkage between mTOR and HIF-1 in PC-3 prostate cancer cells treated during hypoxia was further explored [262]. Pretreatment of PC-3 cells with the mTOR inhibitor, rapamycin, inhibited both the accumulation of HIF-1α and HIF-1-dependent transcription induced by hypoxia [262]. Further work pinpointed the oxygen-dependent degradation domain as a critical target for the rapamycin-sensitive, mTOR-dependent signaling pathway leading to HIF-1α stabilization by hypoxia-inducing agents [262]. These studies position mTOR as an upstream activator of HIF-1 function in cancer cells and suggest that the anti-tumor activity of rapamycin is mediated, in part, through the inhibition of cellular responses to hypoxic stress [262].

Tumor angiogenesis is postulated to be regulated by the balance between pro- and anti-angiogenic factors. It was demonstrated that the critical step in establishing the angiogenic capability of human cells is the repression of the critical anti-angiogenic factor, thrombospondin-1 (Tsp-1) [263]. This repression is essential for tumor formation by mammary epithelial cells and kidney cells engineered to express SV40 early region proteins, hTERT, and H-RasV12. It was demonstrated that Ras induces the sequential activation of PI3K, Rho, and ROCK, leading to activation of Myc through phosphorylation [263]. Phosphorylation of Myc *via* this mechanism enables it to repress Tsp-1 expression [263]. Thus a novel mechanism by which the cooperative activity of the oncogenes, Ras and Myc, leads directly to angiogenesis and tumor formation was described [263].

Over-expression of the v-P3k protein or of cellular PI3K equipped with a myristylation signal, Myr-P3k, induced angiogenesis in the chorioallantoic membrane (CAM) of the chicken embryo [264]. Over-expression of the myristylated form of the PI3K Akt (Myr-Akt) also induces angiogenesis [264]. Over-expression of the tumor suppressor PTEN or of dominant-negative constructs of PI3K inhibited angiogenesis in the yolk sac of chicken embryos, suggesting that PI3K and Akt signaling is required for normal embryonal angiogenesis. The levels of mRNA for VEGF were elevated in cells expressing activated PI3K or Myr-Akt [264]. In human prostate cancer cells, basal-, growth factor-, and mitogen-induced expression of HIF-1α, was blocked by LY294002 and rapamycin [255]. HIF-1-dependent gene transcription was blocked by dominant-negative Akt or PI3K and by wild-type PTEN, whereas transcription was stimulated by constitutively active Akt or dominant-negative PTEN [255]. These data indicated that pharmacological agents that target PI3K, Akt, or mTOR/FRAP in tumor cells inhibit HIF-1α expression and that such inhibition may contribute to therapeutic efficacy. In glioblastoma cells, transcriptional regulation of the VEGF promoter by EGFR was reported to involve Ras/PI3K but to be distinct from signals induced by hypoxia [265]. In breast cancer cell lines LY294002 inhibited HIF-1α induction and phosphorylation under hypoxia [266]. Basal and hypoxia-inducible VEGF expression was reduced at both mRNA and protein levels [266]. V12-Ras overexpression resulted in an increase in hypoxia-induced HIF-1α, which was blocked by the PI3K inhibitor, demonstrating one mechanism for Ras synergy with hypoxia-mediated induction of genes [266]. The decreased HIF-1α expression was not dependent on VHL interaction [266]. Results from another study indicated that HER2 can induce HIF activation *via* the activation of Akt suggesting that activation of HER2/Akt pathway may promote angiogenesis independent of hypoxia, which may have important implications for the oncogenic activity of HER2 and Akt [267].

To genetically test the relationship between HIF-1 and Akt, activated Akt was expressed in a hepatoma cell line lacking HIF-1 [268]. Akt expression was associated with a dramatic increase in tumor size, despite the absence of HIF-1 [268]. Tumor size was not further increased in cells with reconstituted HIF-1 activity, indicating that the effects of Akt on tumorigenesis were not limited by the absence of HIF-1 [268]. Increased tumor size in Akt-expressing, HIF-deficient cells was associated with VEGF secretion and tumor vascularization. Thus, Akt also has potent, HIF-1-independent oncogenic and angiogenic activities [268].

Another study used the Cre-loxP system to generate an endothelial cell-specific mutation of *PTEN* in mice [269]. The gene-targeted mice displayed enhanced tumorigenesis due to an increase in angiogenesis driven by vascular growth factors, an effect which was partially dependent on the p85α and p110γ PI3K isoforms [269]. A critical role for PTEN/PI3K in tumor angiogenesis was thus confirmed [269].

## PI3K SIGNALING IN AUTOPHAGY

Autophagy is a vacuolar, self-digesting mechanism responsible for the removal of long-lived proteins and damaged organelles by the lysosome [270, 271]. The discovery of the autophagy (Atg) genes has provided key information about the formation of the autophagosome, and about the role of macroautophagy in allowing cells to survive during nutrient depletion and/or in the absence of growth factors [270, 271]. Two connected signaling pathways encompassing class I PI3K and mTOR play a central role in controlling macroautophagy in response to starvation [271]. However, a considerable body of literature reports that macroautophagy is also a cell death mechanism that can occur either in the absence of detectable signs of apoptosis (*via* autophagic cell death) or concomitantly with apoptosis [271]. Macroautophagy is activated by signaling pathways that also control apoptosis [271].

It was shown that rapamycin induced autophagy but not apoptosis in rapamycin-sensitive malignant glioma cells by inhibiting the function of mTOR [272]. In contrast, in rapamycin-resistant glioma cells, the inhibitory effect of rapamycin was minor, although the phosphorylation of p70(S6K) was inhibited [272]. Interestingly, LY294002 or an Akt inhibitor both synergistically sensitized rapamycin-sensitive and insensitive cells to rapamycin by stimulating the induction of autophagy [272].

Multiple class III PI3K Vps34p-Vps15p complexes associated with specific regulatory proteins were described and shown to be involved in membrane trafficking events at different sites, with functions in autophagy and carboxypeptidase Y (CPY) sorting [273]. Beclin is involved in the process of autophagy. It was shown that Beclin was co-immunoprecipitated with Vps34p, which is also required for autophagy [274], suggesting that Beclin is a component of the Vps34p complex [275]. Vps34p was shown to regulates carboxypeptidase Y sorting, the constitutive autophagy involving the cytoplasm-to-vacuole targeting (Cvt) and macroautophagy pathways through distinct sets of PI(3)P-binding effectors and that Vps34p promotes protein trafficking in the Cvt pathway through activation/localization of the effector protein Etf1 [276].

## THE ONCOGENIC POTENTIAL OF PI3K

The oncogenic potential of PI3K was first described by studies on ASV 16, a retrovirus that induces hemangiosarcomas in chickens. Analysis of the ASV 16 genome revealed that it encodes an oncogene that is derived from the cellular gene for the catalytic subunit of PI3K. The gene is referred to as v-p3k, and like its cellular counterpart c-p3k, it is a potent transforming gene in cultured chicken embryo fibroblasts (CEFs) [277]. Certain mutated forms of Akt induced oncogenic transformation in chicken embryo fibroblast cultures and hemangiosarcomas in young chickens [278]. This ability to transform cells depends on localization of Akt at the plasma membrane and on the kinase activity of Akt [278]. A transdominant negative form of Akt interfered with oncogenic transformation induced by the p3k oncogene [278]. Akt was therefore shown to be an essential mediator of p3k-induced oncogenicity [278]. The transformed cells showed constitutive phosphorylation of p70(S6K) and of the eukaryotic initiation factor 4E-BP1 binding protein (4E-BP1) [279]. Rapamycin effectively blocked oncogenic transformation induced by either p3k or Akt, in accord with the hypothesis that transformation by p3k or Akt involves mTOR and intervention in translational controls [279].

The ability of activated Ras to stimulate PI3K in addition to Raf was important in Ras transformation of mammalian cells and essential in Ras-induced cytoskeletal reorganization [280]. Further studies showed that PI3K promotes anchorage-independent cell growth, entry into the cell cycle and that prolonged PI3K activation resulted in cellular changes that resemble those associated with oncogenic transformation [281]. Ras oncogene activation induces a proliferative phenotype in normal human thyroid epithelial cells *in vitro*, consistent with its putative role in tumor initiation [282]. In this model, it was shown that PI3K is an absolute requirement for the proliferative response to Ras in these cells, acting *via* suppression of Ras-induced apoptosis [283]. IRS-1, but not Shc, in combination with v-Ha-Ras generates a fully transformed phenotype in 32D cells [284]. Furthermore, transformed 32D/IRS1/Ras cells displayed high levels of PI3K activation and underwent rapid apoptosis when exposed to PI3K inhibitors [284]. Another study described an important role for PI3K/Akt in Ras-mediated transformation of intestinal epithelial cells [285]. Carcinogenesis by oncogenic Ras and Her-2 involves enhanced proliferation of epithelial cells *in vivo*. It was demonstrated that oncogenic H-Ras or constitutively active Her-2 cause increased proliferation and cyclin D1 upregulation in fully polarized, mammary epithelial cells (EpH4), if cultivated as organotypic structures in three-dimensional collagen/matrigel matrices [286]. It was shown that the Ras-activated PI3K pathway is required to induce rapid tumor growth and enhanced proliferation of EpH4 cells in collagen gels, but fails to cause EMT *in vitro* and *in vivo* [286]. Another report suggested that Ras may only affect PI3K signaling when mutationally activated, such as in Ras(V12)-transformed cells, providing a basis for understanding the synergy between Ras and other growth-promoting oncogenes in cancer [287]. In human mammary epithelial cells (HMECs) expressing elevated c-Myc, activated H-Ras is dispensable for anchorage-independent growth. Using this system, it was shown that SV40 small t antigen (st) activates the PI3K pathway and that constitutive PI3K signaling substitutes for st in transformation [288]. Moreover, using constitutively active versions of Akt1 and Rac1, it was shown that these downstream pathways of PI3K synergize to achieve anchorage-independent growth [288]. At lower levels of c-Myc expression, activated PI3K also replaces st to complement H-RasV12 and SV40 large T antigen (LT) and confers both soft agar growth and tumorigenicity [288]. These observations defined the pathways perturbed during the transformation of HMECs [288].

PI3K and some of its downstream targets, such as Akt and p70(S6K) are crucial effectors in oncogenic protein-tyrosine kinase signaling [289]. The ability of the BCR-ABL tyrosine kinase fusion protein to transform hematopoietic cells required PI3K/Akt [290, 291]. BCR/ABL suppresses p27(Kip1) protein levels through PI3K/Akt, leading to accelerated entry into S phase [292]. This activity is likely to explain in part previous studies showing that activation of PI3K was required for optimum transformation of hematopoietic cells by BCR/ABL *in vitro* and *in vivo* [292]. It was also shown that inhibition of p27(Kip1) transcription through PI3K/Akt involves phosphorylation of the forkhead transcription factor FKHR-L1 [293]. In addition, an ubiquitin E3 ligase, the SCF(SKP2) complex, mediates p27(Kip1) ubiquitin-dependent proteolysis. It was shown that SKP2 functions as a critical component in the PTEN/PI3K pathway for the regulation of p27(Kip1) and cell proliferation [294]. BCR-ABL was shown to regulate the cell cycle in CML cells at least in part by inducing proteasome-mediated degradation of the cell cycle inhibitor p27(Kip1), which depends on SKP2 [295]. Further work showed that activation of class I_A_ PI3K and downstream inactivation of FOXO transcription factors are essential for survival of murine pro/pre-B cells transformed by v-ABL or BCR-ABL [296]. In addition, analysis of mice lacking individual PI3K genes indicates that products of the *PIK3R1* gene contribute to transformation efficiency by BCR-ABL [296]. These findings established a role for PI3K signaling in B-lineage transformation by ABL oncogenes.

The most frequently found alteration of the epidermal growth factor receptor (EGFR) in human tumors is a deletion of exons 2-7. This receptor is termed EGFRvIII. High levels of PI3K activity were constitutively present in EGFRvIII-transformed cells and were dependent upon the kinase activity of the receptor. Treatment with the PI3K inhibitors wortmannin and LY294002 blocked both anchorage-independent growth and growth in low serum media and also resulted in morphological reversion of EGFRvIII-transformed cells [47]. Results from another study suggested that the constitutively active EGFRvIII can enhance cell proliferation in glioblastoma in part by down-regulation of p27(Kip1) through activation of the PI3K/Akt pathway [297]. ErbB-2-overexpressing human mammary epithelial (HME) cells exhibit several transformed phenotypes including growth factor independence, anchorage-independent growth, motility, and invasiveness [298]. To identify pathways leading from PI3K to specific phenotypes, constitutively active Akt or PTEN were expressed in erbB-2-overexpressing cells, or in HME cells [298]. HME cells expressing constitutively active Akt were growth factor-independent, anchorage-independent and motile, but not invasive [298]. PTEN expression blocked erbB-2-mediated invasion but none of the other phenotypes [298]. This study concluded that a PI3K-dependent and p38MAPK-dependent pathways lead to activation of Akt, and activation of PKCδ, *via* PI3K, mediates invasion [298]. Breast cancer cells over-expressing ErbB2 depend on its activity for proliferation, because treatment of these cells with ErbB2-specific antagonistic antibodies or kinase inhibitors blocks tumor cells in the G1 phase of the cell cycle. ErbB3 is a partner for ErbB2 in promoting cellular transformation [299]. Loss of functional ErbB2 or ErbB3 had similar effects on cell proliferation and cell cycle regulators [299]. Furthermore, expression of constitutively active Akt rescued the proliferative block induced as a consequence of loss of ErbB2 or ErbB3 signaling [299]. These results demonstrated that ErbB2 over-expression and activity alone are insufficient to promote breast tumor cell division. ErbB3's role is to couple active ErbB2 to the PI3K/Akt pathway [299]. Thus, the ErbB2/ErbB3 dimer functions as an oncogenic unit to drive breast tumor cell proliferation [299].

Polyomavirus (PyV) middle T antigen (MT)-mediated tumorigenesis required activation of both Shc and PI3K, which appeared to be required for stimulation of cell proliferation and survival signaling pathways, respectively [300, 301]. In mammary epithelium, it was reported that activation of Akt can contribute to tumor progression by providing an important cell survival signal but does not promote metastatic progression [302]. Transgenic mice expressing constitutively active Akt were generated. Although expression of activated Akt interfered with normal mammary gland involution, tumors were not observed in these strains [302]. However, coexpression of activated Akt with a mutant middle T antigen decoupled from PI3K (MTY315/322F) resulted in a dramatic acceleration of mammary tumorigenesis correlated with reduced apoptotic cell death [302]. Furthermore, co-expression of activated Akt with MTY315/322F resulted in phosphorylation of the FKHR transcription factor and translational up-regulation of cyclin D1 levels [302].

Transformation of chicken embryo fibroblasts (CEF) by v-Src was mediated by two parallel pathways, the Ras-MAPK pathway and the PI3K-mTOR pathway, which both contributed to transformation [303]. v-Src induced constitutive activation of phosphatidylinositol 3-kinase led to factor-independent proliferation [304]. A dominant-negative mutant of PI3K (Deltap85) partially inhibited v-Src-dependent growth [304]. MEN2A and FMTC mutations result in a constitutive catalytic activity of the RTK RET and as a consequence convert RET into a dominantly acting transforming gene. The mutant RET-mediated cell-transforming effect was shown to be critically dependent on the activation of the PI3K/AKT pathway [305]. Membrane localization of the the constitutively activated Tpr-Met oncoprotein enhanced cellular transformation, focus formation, and anchorage-independent growth and induces tumors with a distinct myxoid phenotype [306]. This correlated with the induction of hyaluronic acid (HA) and the presence of a distinct form of its receptor, CD44 [306]. A pharmacological inhibitor of PI3K inhibited the production of HA, and conversely, an activated, plasma membrane-targeted form of PI3K was sufficient to enhance HA production [306]. These results provided a positive link to a role for HA and CD44 in Met receptor-mediated oncogenesis and implicated PI3K in these events [306]. v-Crk induces cellular tyrosine phosphorylation and transformation of chicken embryo fibroblasts (CEF). Constitutive activation of the PI3K/Akt pathway by v-Crk was shown to play an essential role in v-Crk-induced transformation of CEF [307]. A subsequent report suggested the involvement of the PI3K/Akt survival pathway in the v-Crk-induced protection against apoptosis [308]. NPM/ALK encodes a constitutively activated tyrosine kinase that belongs to the family of tyrosine kinases activated by the chromosomal translocation t(2;5) in a subset of anaplastic large cell lymphomas. It was reported that NPM/ALK constitutively activates the PI3K-Akt pathway and that this pathway plays an important role in the NPM/ALK-mediated malignant transformation [309]. Studies with a PI3K inhibitor and a c-Kit mutant incapable of recruiting PI3K, indicated that constitutive activation of PI3K through direct recruitment by constitutively active D816V c-Kit plays a role in factor-independent growth of immortalized murine progenitor cells and is critical for tumorigenicity [310].

To determine the specific functions of p110α, mice carrying a conditionally targeted allele of the *PIK3CA* gene were generated [311]. *PIK3CA*-knockout mouse embryonic fibroblasts were deficient in cellular signaling in response to various growth factors and resistant to oncogenic transformation induced by a variety of oncogenic receptor tyrosine kinases, indicating a fundamental role for p110α in these biological processes [311]. In a recent study, mice with mutations in the *PIK3CA* gene that block its interaction with Ras were generated [312]. Cells from these mice showed proliferative defects and selective disruption of signaling from growth factors to PI3K. Importantly, the mutant mice were highly resistant Ras oncogene-induced tumorigenesis [312]. The interaction of Ras with p110α was thus demonstrated to be required *in vivo* for normal growth factor signaling and for Ras-driven tumor formation [312].

## ALTERATIONS IN PI3K SIGNALING IN CANCER

A summary of the main genetic alterations described in the PI3K signaling pathway is presented in Table **1**. We will first discuss the evidence describing altered PI3K signaling in human cancer, before more specifically addressing alterations in *PTEN*, *PIK3CA* and the p85 regulatory subunit of PI3K.

## ACTIVATION OF THE PI3K/AKT SIGNALING PATHWAY IN HUMAN CANCER

A first study showed that colorectal tumors exhibited enhanced PI3K activity compared with normal colonic mucosa, raising the possibility that PI3K may be a potential target for new strategies for the treatment of colorectal carcinoma [313]. In small cell lung cancer (SCLC) initial reports found high basal constitutive PI3K activity, which results in high basal Akt and ribosomal p70(S6K) activity [314]. Inhibition of PI3K activity markedly inhibited SCLC cell proliferation in liquid culture as a result of stimulating apoptosis and promoting cell cycle delay in G1 [314]. Thus, constitutive PI3K activity in SCLC cells was proposed to play an important role in promoting the growth and anchorage independence of SCLC [314]. Another study identified Akt as a constitutively active kinase that promotes survival of NSCLC cells and demonstrated that modulation of Akt activity by pharmacological or genetic approaches alters the cellular responsiveness to therapeutic modalities such as chemotherapy or radiotherapy [315]. Elevated phospho-Akt staining was reported in 65% human malignant mesotheliomas (MM) specimens [316]. In addition, Akt phosphorylation was consistently observed in MMs arising in asbestos-treated mice and in MM cell xenografts [316]. Treatment of a MM cell line with rapamycin resulted in growth arrest in G1 phase, while LY294002 in combination with cisplatin had greater efficacy in inhibiting cell proliferation and inducing apoptosis than either agent alone [316]. 

A report showed activation of Akt2 in human primary ovarian cancer and induction of apoptosis by inhibition of PI3K/Akt pathway [317]. The majority of tumors displaying activated Akt2 were high grade and stages III and IV [317]. Immunostaining and Western blot analyses using a phospho-Ser-473 Akt antibody that detects the activated form of Akt2 confirmed the frequent activation of Akt2 in ovarian cancer specimens [317]. Another study determined the frequency of Akt activation in ovarian cancer and found elevated staining (phosphor-Ser473) in 68% ovarian carcinomas [318]. In another report, significantly increased Akt1 kinase activity was detected in primary carcinomas of prostate, breast, and ovary [319]. The majority of Akt1-activated tumors were high grade and stage III/lV [319]. 

In thyroid cancer, increased levels of phosphorylated total Akt were identified in follicular but not papillary cancers compared with normal tissue [320]. Levels of Akt1 and Akt2 proteins and Akt2 RNA were elevated only in the follicular cancers [320]. In paired samples, Akt 1, 2, 3, and phospho-Akt levels were higher in cancers [320]. These data suggested that Akt activation may play a role in the pathogenesis or progression of sporadic thyroid cancer [320]. In head and neck cancer, a significant association was found between phospho-Akt staining and local recurrence in the patient series. Evaluation of PI3K activation by Akt phosphorylation was thus suggested to be a prognostic marker for response to therapy [321]. Immunohistochemical analyses in breast carcinomas revealed that elevated expression of HER-2/neu was found to correlate with over-expression of Akt2 protein and activation of Akt kinase [322]. HER-2/neu-overexpressing breast cancer cell lines were resistant to apoptosis induced by UV treatment and hypoxia, which was suppressed in the presence of the PI3K inhibitors LY294002 and wortmannin, indicating a link between Akt activation and stress resistance in HER-2/neu-overexpressing cells [322]. In colorectal carcinomas, immunohistochemical analysis showed that 46% of the tumors had a high level of expression of phosphorylated Akt with a close association with Ki-67 proliferative activity and the number of apoptotic bodies [323]. Akt phosphorylation was also correlated with clinicopathologic parameters of the malignancies, including depth of invasion, infiltration to venous vessels, lymph node metastasis, and clinicopathologic stage [323]. It was concluded that activation of Akt plays an important role during the progression of colorectal carcinomas by helping promote cell growth and rescue cells from apoptosis [323]. 

Another study suggested that PI3K has a major role in the control of proliferation and apoptosis of growth factor-independent multiple myeloma cell lines [324]. Constitutive activation of this pathway was shown to be a frequent event in the biology of multiple myeloma *in vivo* and may be more frequently observed in primary plasma cell leukemia [324]. Purified plasmocytes from patients with myeloma or leukemia displayed constitutive phosphorylation of Akt, FKHRL-1 and p70(S6K), which was inhibited by LY294002 and enhanced by IGF-I [324]. Another study demonstrated that Akt is activated in AML blasts and that p70(S6K) and 4EBP-1, downstream mediators of Akt signaling, also are phosphorylated in AML blasts [325]. In a short-term culture system, most AML patient samples showed a dose-dependent decrease in survival after incubation with LY294002 [325]. Incubation of AML blasts with RAD001 induced only a small decrease in survival of the cells [325]. However, when combined with Ara-C, RAD001 enhanced the toxicity of Ara-C [325]. These results demonstrated that constitutive activation of the PI3K pathway is necessary for the survival of AML blasts and that targeting of this pathway with pharmacologic inhibitors may be of clinical benefit in treatment of AML [325]. Another study demonstrated that the overall survival of patients with the Akt phosphoryated on Ser473 was significantly shorter than that of patients without [326]. Thus, the detection of the Akt phosphorylation may provide a new tool for identifying AML patients at high risk of an unfavorable outcome [326].

Based on the observation that melanoma cell lines exhibit constitutive Akt activation, this event was evaluated by immunohistochemistry [327]. Normal and slightly dysplastic nevi exhibited no significant Akt expression, in marked contrast to the dramatic Akt immunoreactivity seen in severely dysplastic nevi and melanomas [327]. It was proposed that activation of Akt may be an early marker for tumor progression in melanoma [327].

In glioma, a study analysed the levels of expression of PI3K pathway members through quantitative Western analysis [328]. Levels of phospho-Akt, and phospho-p70(S6K) were all found to be inversely associated with cleaved caspase-3 levels, suggesting PI3K pathway activation is associated with reduced levels of apoptosis [328]. Activation of PI3K pathway members was found to be significantly associated with reduced survival times [328]. 

A study examined the status of activation of Akt in different stages of squamous cell carcinoma development in clinical samples from squamous carcinomas of the head and neck (HNSCC) patients [329]. By immunohistochemical analysis, it was demonstrated that activation of Akt is a frequent event in human HNSCC because active Akt could be detected in these tumors with a pattern of expression and localization correlating with the progression of the lesions [329]. In line with these observations, Akt was constitutively activated in a large fraction of HNSCC-derived cell lines [329].

Gefitinib, a specific epidermal growth factor receptor (EGFR) tyrosine kinase inhibitor, was shown to have activity against approximately 10% of unselected non-small-cell lung cancer (NSCLC) patients. An important finding was that patients with phosphorylated Akt-positive tumors who received gefitinib had a better response rate, disease control rate, and time to progression than patients with phosphorylated Akt-negative tumors, suggesting that gefitinib may be most effective in patients with basal Akt activation [330].

## THE TUMOR SUPPRESSOR PTEN

### Discovery of PTEN as an Antagonist of the PI3K/Akt Pathway

The tumor suppressor called phosphatase and tensin homolog deleted on chromosome 10 (PTEN) (or MMAC1) is located on human chromosome 10q23, and was initially described as sharing homology with the protein tyrosine phosphatase family [331, 332]. Germline mutations in *PTEN* give rise to several related neoplastic disorders, including Cowden disease [331, 333, 334]. It was demonstrated that over-expression of PTEN reduced insulin-induced PIP_3_ production in human cells without effecting insulin-induced PI3K [335]. Purified recombinant PTEN catalyzed dephosphorylation of PIP_3_, specifically at position 3 on the inositol ring [335]. These results established the function of PTEN as a phosphoinositide 3-phosphatase by regulating PIP_3_ levels [335]. Although more than half of *PTEN* mutations result in protein truncation, a significant fraction of *PTEN* mutations are missense mutations. It was shown that the majority of *PTEN* missense mutations (90%) eliminated or reduced phosphatase activity towards inositol 1,3,4,5-tetrakisphosphate and PIP_3_ [336]. It was reported that a missense mutation in *PTEN*, PTEN-G129E, which is observed in two Cowden disease kindreds, specifically ablates the ability of PTEN to recognize inositol phospholipids as a substrate, suggesting that loss of the lipid phosphatase activity is responsible for the etiology of the disease [337]. Furthermore, expression of wild-type or substrate-trapping forms of PTEN in mammalian cells altered the levels of the phospholipid products of PI3K and ectopic expression of the phosphatase in PTEN-deficient tumor cell lines resulted in the inhibition of PKB/Akt and regulation of cell survival [337]. It was also shown that glioblastoma cells, in contrast to primary human astrocytes, contain high endogenous Akt activity and high levels of PI(3,4,5)P_3_ and PI(3,4)P_2_, the lipid products of PI3K [338]. These glioblastoma cells were shown to express mutant forms of PTEN [338]. PTEN antagonized the activation of PKB/Akt by growth factors, by activated PI3K and by PDK1, but did not antagonize the phospholipid-independent activation of PKB/Akt lacking the PH domain [338]. These results confirmed a role for PTEN in regulating the activity of the PI3K pathway in malignant human cells. Another study demonstrated that the acute administration of MMAC/PTEN in glioma cells infected with recombinant adenoviruses resulted in the inhibition of Akt-mediated signaling, growth inhibition, and anoikis [339]. In another report, PTEN inhibited cell growth and/or colony formation in epithelial cell lines [340]. The decrease in cellular proliferation was associated with an induction of apoptosis and an inhibition of signaling through the PI3K pathway [340]. Akt/ PKB was able to rescue cells from PTEN-dependent death [340]. PTEN expression potently suppressed the growth and tumorigenicity of human glioblastoma U87MG cells [341]. The growth suppression activity of PTEN was mediated by its ability to block cell cycle progression in the G1 phase [341]. Such an arrest correlated with a significant increase of the cell cycle kinase inhibitor p27(KIP1) and a concomitant decrease in the activities of the G1 cyclin-dependent kinases [341]. PTEN expression also led to the inhibition of Akt/ PKB [341]. Further work implicated p27(KIP1) as a critical mediator of PTEN-induced G1 arrest [342]. It was also shown that PTEN protein induces a G1 block when reconstituted in PTEN-null cells [343]. A PTEN mutant associated with Cowden's disease (PTEN;G129E) has protein phosphatase activity yet is defective in dephosphorylating inositol 1,3,4,5-tetrakisphosphate *in vitro* and fails to arrest cells in G1 [343]. These data suggest a link between induction of a cell-cycle block by PTEN and its ability to dephosphorylate, in *vivo*, PIP_3 _[343]. In a parallel study, PTEN impaired activation of endogenous Akt in cells and inhibited phosphorylation of 4E-BP1 [344]. In addition, PTEN/ MMAC1 repressed gene expression in a manner that is rescued by Akt but not PI3K [344]. Finally, higher levels of Akt activation are observed in human prostate cancer cell lines and xenografts lacking PTEN/MMAC1 expression when compared with PTEN/MMAC1-positive prostate tumors or normal prostate tissue [344]. 

### Animal Models

PTEN-mutant mouse embryos displayed regions of increased proliferation [345]. In contrast, PTEN-deficient immortalized mouse embryonic fibroblasts exhibited decreased sensitivity to cell death in response to a number of apoptotic stimuli, accompanied by constitutively elevated activity and phosphorylation of PKB/Akt [345]. Expression of exogenous PTEN in mutant cells restores both their sensitivity to agonist-induced apoptosis and normal pattern of PKB/Akt phosphorylation. Furthermore, PTEN negatively regulated intracellular levels of PIP_3_ in cells and dephosphorylates it *in vitro *[345]. These results showed that PTEN may exert its role as a tumor suppressor by negatively regulating the PI3K/PKB/Akt signaling pathway. The *PTEN *gene was shown to be fundamental for embryonic development in mice, as *PTEN* mutant embryos died by day 9.5 of gestation [346]. Heterozygous mice developed lymphomas associated with loss of heterozygosity of the wild-type *PTEN* allele, and tumor appearance was accelerated by gamma-irradiation [346]. These lymphomas had high levels of activated Akt/PKB [346]. This suggested that tumors associated with *PTEN* loss of heterozygosity may arise as a consequence of an acquired survival advantage [346]. When more than 6 months old, *PTEN*(+/-) mice were shown to develop a range of tumors, partially resembling the spectrum of neoplasia observed in Cowden's syndrome patients [347]. One-half of *PTEN* (+/-) females developed breast tumors, whereas all of the females had endometrial hyperplasia, and there was a high incidence of endometrial cancer [347]. Analysis of prostate cancer progression in transgenic adenocarcinoma of mouse prostate mice bred to *PTEN*(+/-) heterozygous mice, coupled with analysis of the PTEN gene and protein in the resulting tumors, revealed that haploinsufficiency of the *PTEN* gene promotes the progression of prostate cancer in this model system [348]. Using the Cre-loxP system, another group selectively inactivated *PTEN *in skin and prostate in mice [349]. Abnormalities in *PTEN* mutant skin consisted of mild epidermal hyperplasia, whereas prostates from these mice exhibited high-grade prostatic intraepithelial neoplasia that frequently progressed to focally invasive cancer [349]. These data demonstrated that PTEN is an important physiological regulator of growth in the skin and prostate [349]. Further, the early onset of prostatic neoplasia in *PTEN* mutant males implicated *PTEN* mutations in the initiation of prostate cancer. Consistent with high *PTEN* mutation rates in human prostate tumors, these data indicated that PTEN is a critical tumor suppressor in this organ.

Primordial germ cells (PGCs), which are the embryonic precursors of gametes, are the source of testicular teratoma. To elucidate the intracellular signaling mechanisms that underlie germ cell differentiation and proliferation, mice with a PGC-specific deletion of the *PTEN* gene were generated [350]. Male mice that lacked *PTEN *exhibited bilateral testicular teratoma, which resulted from impaired mitotic arrest and outgrowth of cells with immature characters [350]. Experiments with PTEN-null PGCs in culture revealed that these cells had greater proliferative capacity and enhanced pluripotent embryonic germ cell colony formation [350]. PTEN thus appears to be essential for germ cell differentiation and an important factor in testicular germ cell tumor formation [350]. 

In another study aimed at determining the role of the PI3K pathway in pancreas development, a pancreas-specific knockout of *PTEN* was generated [351]. Knockout mice displayed progressive replacement of the acinar pancreas with highly proliferative ductal structures that contained abundant mucins and expressed markers of pancreatic progenitor cells [351]. Moreover, a fraction of these mice develop ductal malignancy [351]. Thus, misregulation of the PI3K pathway may contribute to the initiation of pancreatic carcinoma *in vivo* [351].

### Functional Consequences of PTEN Inactivation

A null mutation was introduced into the mouse *PTEN* gene by homologous recombination in embryonic stem (ES) cells [352]. *PTEN* (-/-) ES cells exhibited an increased growth rate and proliferated even in the absence of serum. ES cells lacking PTEN function also displayed advanced entry into S phase [352]. This accelerated G1/S transition was accompanied by down-regulation of p27(KIP1), a major inhibitor for G1 cyclin-dependent kinases [352]. Inactivation of *PTEN* in ES cells and in embryonic fibroblasts resulted in elevated levels of PIP_3_. Consequently, *PTEN* deficiency led to dosage-dependent increases in phosphorylation and activation of Akt/PKB and Akt activation increased Bad phosphorylation and promoted *PTEN* (-/-) cell survival [352]. Fas-mediated apoptosis was impaired in *PTEN* (+/-) mice, and T lymphocytes from these mice show reduced activation-induced cell death and increased proliferation upon activation. PI3K inhibitors restored Fas responsiveness in *PTEN* (+/-) cells [353]. These results indicated that PTEN is an essential mediator of the Fas response and a repressor of autoimmunity and thus implicated PI3K/Akt pathway in Fas-mediated apoptosis [353].

PTEN was shown to suppress breast cancer growth through down-regulating PI3K signaling, which leads to the blockage of cell cycle progression and the induction of cell death in a sequential manner [354]. In breast cancer cells under anchorage-independent conditions, PTEN also induced anoikis, a form of apoptosis that occurs when cells are dissociated from the extracellular matrix, which is enhanced in conjunction with low serum culture conditions [355]. Together, these data suggest that PTEN effects on the PI3K signaling cascade are influenced by the cell stimulatory context, and that depending on the exposure to growth factors and other exogenous stimuli such as integrin ligation, PTEN can induce cell cycle arrest, apoptosis or anoikis in breast cancer cells. The tumour suppressor activities of PTEN were linked to the machinery controlling cell cycle through the modulation of signaling molecules whose final target is the functional inactivation of the retinoblastoma gene product [356]. Expression of wild-type PTEN reduced the expression of cyclin D1 [357]. Cyclin D1 reduction was accompanied by a marked decrease in endogenous retinoblastoma (Rb) protein phosphorylation on cyclin D/CDK4-specific sites, showing an early negative effect of PTEN on Rb inactivation. PTEN expression also prevented cyclin D1 from localizing to the nucleus during the G(1)- to S-phase cell cycle transition [357]. The PTEN-induced localization defect and the cell growth arrest could be rescued by the expression of a nucleus-persistent mutant form of cyclin D1, indicating that an important effect of PTEN is at the level of nuclear availability of cyclin D1 [357]. Furthermore, in human glioblastoma cells, *PTEN* mutation can cooperate with EGFR activation to increase VEGF mRNA levels by transcriptionally up-regulating the proximal VEGF promoter *via* the PI3K/Akt pathway [358]. In addition, the ability of PTEN to potently inhibit H-Ras-induced morphological transformation and anchorage-independent growth in NIH3T3 cells was reported [359]. It was also shown that PTEN can regulate prostate cancer cell proliferation and apoptosis through inhibition of IGF-IR synthesis [360]. 

### Clinical Findings

Loss of PTEN protein was reported to correlate with pathological markers of poor prognosis in prostate cancer [361]. A relative reciprocity of mutations in *PTEN *and *NRAS* was also reported in melanoma, suggesting that the two genetic changes, in a subset of cutaneous melanomas, are functionally overlapping [362]. In multiple myeloma (MM) it was shown that human lines possessing the highest Akt activity lost PTEN expression [363]. Sequencing analysis demonstrated that the *PTEN* gene contained a deletion. Restoration of PTEN expression suppressed IGF-I-induced Akt activity, suggesting that loss of PTEN is responsible for uncontrolled Akt activity in MM lines [363]. In lymphoma/leukemia-derived cell lines, an inverse relationship between PTEN and phosphorylated Akt was observed in 63% of cell lines [364]. No cell lines showed absence of PTEN expression, whereas 50% of cell lines showed low PTEN expression [364]. Another study demonstrated the phosphorylation of Akt is accompanied by the loss of PTEN in clinical specimens of endometrial carcinomas [365]. Phosphorylation of Akt was accompanied by the loss of PTEN in clinical specimens of endometrial cancers [366]. The survival rate for PTEN-positive patients was significantly higher than that for PTEN-negative or -heterogeneous staining patients, and thus PTEN-positive staining was proposed to be a significant prognostic indicator of favorable survival for patients with advanced endometrial cancer [366]. In glioma, a strong inverse correlation was described between PTEN levels and both phosphorylated Akt expression and Akt activity [367]. A significant association was evident between PTEN expression level and histology. PTEN levels were highest in normal brain, lowest in GBM tumors, and intermediate in grade II oligoastrocytomas [367]. Another study demonstrated that loss PTEN was highly correlated with activation of the main PI3K effector Akt *in vivo* [368]. It was also shown that Akt activation is significantly correlated with mTOR, the family of forkhead transcription factors (FOXO1, FOXO3a, and FOXO4) and the S6 protein [368]. Expression of the mutant EGFRvIII was also tightly correlated with phosphorylation of these effectors, demonstrating an additional route to PI3K pathway activation in glioblastomas *in vivo* [368]. PTEN expression correlated significantly with survival time within the entire cohort and was associated with survival within the subgroup of GBM tumors [367]. Thus, reduced PTEN expression is ubiquitous among GBM tumors and may play a role in the development of low-grade gliomas. PTEN inactivation in gliomas portends a particularly aggressive clinical behavior [367].

### Therapeutic Implications

It was shown that transformed cells of *PTEN* (+/-) mice have elevated levels of phosphorylated Akt and activated p70(S6K) associated with an increase in proliferation. Pharmacological inactivation of mTOR/RAFT/FRAP reduced neoplastic proliferation, tumor size, and p70(S6K) activity, but did not affect the status of Akt [369]. These data suggested that p70(S6K) and possibly other targets of mTOR contribute significantly to tumor development and that inhibition of these proteins may be therapeutic for cancer patients with deranged PI3K signaling [369]. *In vitro* and *in viv*o studies of isogenic *PTEN* (+/+) and *PTEN* (-/-) mouse cells as well as human cancer cells with defined *PTEN* status confirmed that the growth of *PTEN* null cells was blocked preferentially by pharmacologic FRAP/mTOR inhibition [370]. Enhanced tumor growth caused by constitutive activation of Akt in PTEN (-/-) cells also was reversed by CCI-779 (rapamycin derivative) treatment, indicating that mTOR functions downstream of Akt in tumorigenesis [370]. Loss of *PTEN* correlated with increased S6 kinase activity and phosphorylation of ribosomal S6 protein, providing evidence for activation of the FRAP/mTOR pathway in these cells [370]. Another study aimed to determine the effects of *PTEN* status and treatment with rapamycin in the response of prostate cancer cell lines to doxorubicin [371]. The PTEN-positive cells were significantly more susceptible to the anti-proliferative effects of doxorubicin as compared with the PTEN-negative cells. Transfection of PTEN into the PTEN-negative decreased the activation of Akt and the downstream p70(S6K) and reversed the resistance to doxorubicin in these cells, indicating that changes in PTEN status/Akt activation modulate the cellular response to doxorubicin [371]. Treatment of PC-3 PTEN-negative cells with rapamycin inhibited p70(S6K) and increased the proliferative response of these cells to doxorubicin [371]. Furthermore, treatment of mice bearing the PTEN-negative prostate cancer xenografts with CCI-779, an ester of rapamycin combined with doxorubicin, inhibited the growth of the doxorubicin-resistant tumors confirming the observations *in vitro* [371]. Thus, rapamycin and CCI-779, by interacting with downstream intermediates in the PI3K/Akt signaling pathway, reverse the resistance to doxorubicin conferred by PTEN mutation/Akt activation [371]. Another study examined the possible mechanisms of resistance to the EGFR inhibitor ZD1839 (Iressa) in tumor cells with variable levels of EGFR [372]. The results suggested that loss of *PTEN*, by permitting a high level of Akt activity independent of RTK inputs, can temporally dissociate the inhibition of the EGFR with that of Akt induced by EGFR inhibitors [372]. Thus, it was suggested that in EGFR-expressing tumor cells with concomitant amplification(s) of PI3K/Akt signaling, combined blockade of the EGFR tyrosine kinase and Akt should be considered as a therapeutic approach [372]. In another study, sensitivity to ZD1839 required intact growth factor receptor-stimulated Akt signaling activity. PTEN loss leads to uncoupling of this signaling pathway and results in ZD1839 resistance, which could be reversed with reintroduction of PTEN or pharmacologic down-regulation of constitutive PI3K/Akt pathway activity [373].

In myeloma, PI3K inhibitors preferentially suppressed *PTEN*-null myeloma growth to those expressing PTEN, indicating that PI3K activation is more critical for growth and survival of those lines with *PTEN* mutations than others expressing a functional PTEN gene [374]. Expression of an active Akt, reversed wortmannin- and dexamethasone-induced apoptosis and growth inhibition in *PTEN*-null myeloma lines, suggesting that Akt lies downstream of PI3K for PTEN-null myeloma survival and dexamethasone resistance [374].

The ErbB2-targeting antibody, trastuzumab (Herceptin), has remarkable therapeutic efficacy in certain patients with ErbB2-overexpressing tumors. The overall trastuzumab response rate for reasons that are not completely understood. It was reported that PTEN activation contributes to trastuzumab's antitumor activity. Reducing PTEN in breast cancer cells by antisense oligonucleotides conferred trastuzumab resistance *in vitro* and *in vivo *[375]. Patients with *PTEN*-deficient breast cancers had significantly poorer responses to trastuzumab-based therapy than those with normal *PTEN* [375]. Additionally, PI3K inhibitors rescued PTEN loss-induced trastuzumab resistance [375]. Thus, PTEN deficiency was proposed to be a predictor for trastuzumab resistance [375]. 

Forkhead family of transcription factors (FOXO1a, FOXO3a, FOXO4) are downstream targets of the PI3K/PTEN/Akt pathway. In *PTEN* null cells, FOXO1a is inactivated by PI3K-dependent phosphorylation and mislocalization to the cytoplasm, yet still undergoes nucleocytoplasmic shuttling. Since forcible localization of FOXO1a to the nucleus can reverse tumorigenicity of *PTEN *null cells, a high-content, chemical genetic screen for inhibitors of FOXO1a nuclear export was performed [376]. The compounds detected in the primary screen were retested in secondary assays, and structure-function relationships were identified. Novel general export inhibitors were found that react with CRM1 as well as a number of compounds that inhibit PI3K/Akt signaling, among which are included multiple antagonists of calmodulin signaling [376].

### Interactions with Other Tumor Suppressor Pathways

The PTEN tumor suppressor protein inhibits PI3K/Akt signaling that promotes translocation of Mdm2 into the nucleus [195, 377]. When restricted to the cytoplasm, Mdm2 is degraded [377]. The ability of PTEN to inhibit the nuclear entry of Mdm2 increases the cellular content and transactivation of the p53 tumor suppressor protein. Retroviral transduction of PTEN into *PTEN* null glioblastoma cells increases p53 activity and expression of p53 target genes and induces cell cycle arrest [378]. U87MG/PTEN glioblastoma cells were more sensitive than U87MG/PTEN null cells to death induced by etoposide, a chemotherapeutic agent that induces DNA damage [378]. These results established a direct connection between the activities of two major tumor suppressors and show that they act together to respond to stresses and malignancies. PTEN protects p53 from survival signals, permitting p53 to function as a guardian of the genome [377, 378].

### Mutations in Other Inositol Lipid Phosphatases

The SH2 domain-containing inositol 5'-phosphatase (SHIP) is crucial in hematopoietic developement. A somatic mutation at codon 684, replacing Val with Glu, was detected in one acute myeloid leukemia AML patient, lying within the signature motif 2, which is the phosphatase active site [379]. The results of an *in vitro* inositol 5'-phosphatase assay revealed that the mutation reduced catalytic activity of SHIP [379]. Leukemia cells with the mutation showed enhanced Akt phosphorylation following IL-3 stimulation [379]. K562 cells transfected with the mutated SHIP-V684E cDNA showed a growth advantage even at lower serum concentrations and resistance to apoptosis induced by serum deprivation and exposure to etoposide [379]. These results suggest a possible role of the mutated SHIP gene in the development of acute leukemia and chemotherapy resistance through the deregulation of the PI3K/Akt signaling pathway [379]. This was the first report of a mutation in the SHIP gene in any given human cancer, and indicates the need for more attention to be paid to this gene with respect to cancer pathogenesis [379].

## THE HUMAN *PIK3CA *ONCOGENE

### Increases in *PIK3CA* Copy Numbers

Studies using comparative genomic hybridization (CGH) revealed several regions of recurrent, abnormal, DNA sequence copy number that may encode genes involved in the genesis or progression of ovarian cancer [380]. One region at 3q26 found to be increased in copy number in approximately 40% of ovarian and others cancers contains *PIK3CA*, which encodes the p110α catalytic subunit of PI3K [380]. *PIK3CA* was shown to be frequently increased in copy number in ovarian cancers, and the increased copy number is associated with increased *PIK3CA* transcription, p110α protein expression and PI3K activity and the treatment with the PI3K inhibitor LY294002 decreased proliferation and increases apoptosis [380]. These observations suggested *PIK3CA* is an oncogene that has an important role in ovarian cancer [380]. In a subsequent study, *PIK3CA* mRNA was detected in 66.6% of stage I and 93.9% of advanced stage ovarian cancer specimens and in all ovarian cancer cell lines [381]. *PIK3CA* mRNA levels were significantly higher in invasive carcinomas compared with benign and low malignant potential neoplasms [381]. Strong expression of immunoreactive p110α was detected in tumor cells and/or stroma endothelium [381]. *PIK3CA* expression *in vivo* positively correlated, both at the mRNA and the protein level, with the expression of VEGF as well as with the extent of microvascular development [381]. Furthermore, *PIK3CA* mRNA over-expression positively correlated with increased proliferation and decreased apoptosis of tumor cells *in vivo* [381]. *In vitro*, *PIK3CA* expression positively correlated with the expression of VEGF in ovarian cancer cells, whereas LY294002 reduced both the constitutive and inducible expression of HIF-1α at the mRNA and protein levels and abrogated VEGF up-regulation by glucose starvation [381]. Furthermore, LY294002 suppressed cell proliferation and, at higher doses, induced marked apoptosis in ovarian cancer cells. Collectively, these data strongly indicate that *PIK3CA* supports ovarian cancer growth through multiple and independent pathways affecting cell proliferation, apoptosis and angiogenesis, and plays an important role in ovarian cancer progression [381].

The results of CGH also showed that the 3q26.3 amplification was the most consistent chromosomal aberration in primary tissues of cervical carcinoma, and a positive correlation between an increased copy number of *PIK3CA* and 3q26.3 amplification was found in tumor tissues and in cervical cancer cell lines [382]. In cervical cancer cell lines harboring amplified *PIK3CA*, the expression of p110α was increased, and was subsequently associated with high kinase activity [382]. These evidences supported that *PIK3CA* is an oncogene in cervical cancer and *PIK3CA* amplification may be linked to cervical tumorigenesis [382]. In low-grade head and neck squamous cell carcinomas *PIK3CA* was over-represented, as analyzed by CGH or fluorescence in situ hybridization [383]. These results indicated that *PIK3CA* may participate to the progression of head and neck tumors [383].

In another study, *PIK3CA* was identified as an oncogene involved in squamous cell carcinomas [384]. Simultaneous abnormalities in both pathways were rare in primary tumors, suggesting that amplification of *PIK3CA* and mutation of p53 are mutually exclusive events and either event is able to promote a malignant phenotype [384]. Moreover, the negative effect of p53 induction on cell survival involved the transcriptional inhibition of *PIK3CA* that was independent of PTEN activity, as PTEN was not expressed in the primary tumors [384]. Conversely, constitutive activation of *PIK3CA* resulted in resistance to p53-related apoptosis in PTEN deficient cells [384]. Thus, p53 regulates cell survival by inhibiting the PI3K/AKT prosurvival signal independent of PTEN in epithelial tumors. This inhibition is required for p53-mediated apoptosis in malignant cells [384].

Array CGH was used to identify genomic abnormalities at loci encoding genes that may contribute to lung cancer transformation and progression in squamous carcinomas (SqCas) and adenocarcinomas (AdCas) [385]. The most noticeable differences between SqCas and AdCas were gain of chromosome 3q22-q26 and loss of chromosome 3p [385]. These occurred almost exclusively in SqCas and the region of recurrent increase contained *PIK3CA* [385]. The activity of Akt was higher in SqCas than in AdCas and was correlated with *PIK3CA* copy number, suggesting that these copy number increases contribute to activation of PI3K signaling in SqCas of the lung [385]. In head and neck squamous cell carcinoma (HNSCC) a study suggested that 3q26 copy number gain and amplification represent early genomic aberrations in HNSCC carcinogenesis [386]. In addition, p110α mRNA and protein expression in HNSCC may be regulated by these genomic aberrations as well as by epigenetic events [386].

### Discovery of Somatic Mutations in *PIK3CA*

**Fig. (2).**
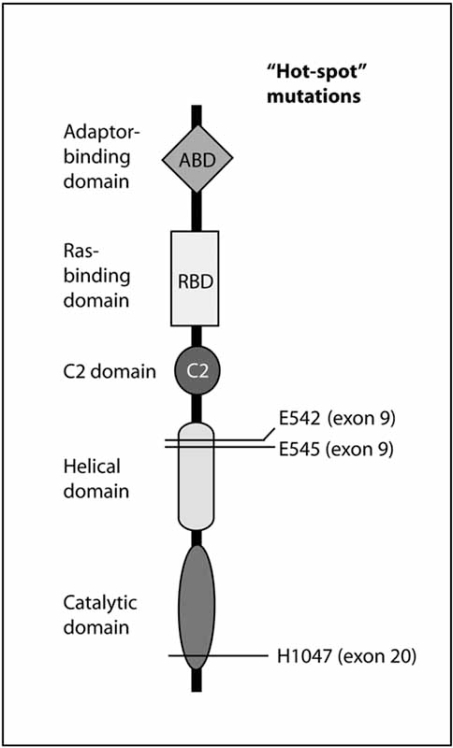
**Schematic of *PIK3CA* structure and “hot-spot” mutations observed in human cancers.** Cancer-specific mutations are clustered in the helical and catalytic domain of p100α.Several mutations have been reported in human solid tumors for *PIK3CA*, the gene that encodes the catalytic subunit p110α  of PI3K. High frequency of missense mutations is observed at the amino acid residues E542, E545 and H1047.

To determine if PI3Ks are genetically altered in tumorigenesis,the PI3K genes were sequenced in human cancers and corresponding normaltissue [387].Eight PI3K and eightPI3K-like genes, including two uncharacterized genes, were identified in thehuman genome [387]. The sequencesof 117 exons that encode the predicted kinase domains of thesegenes were examined in 35 colorectal cancers [387]. *PIK3CA* was the only gene with somatic (i.e.,tumor-specific) mutations [387]. Subsequent sequence analysis of allcoding exons of *PIK3CA* in 199 additional colorectal cancersrevealed mutations in a total of 74 tumors (32%). The authors also evaluated 76 premalignant colorectaltumors and found only two mutations [387]. Thus,*PIK3CA* mutations generally arise late in tumorigenesis, just before or coincident with invasion. Mutations in *PIK3CA* were also identified in glioblastoma, gastric cancer, breast cancer and lung cancer [387]. In total, 92 mutations were observed, all of which were determinedto be somatic in the cancers that could be assessed [387]. No truncating mutations were observed and >75% of alterations occurred in two smallclusters in the helical and kinase domains [387] (Fig. **2**). The affected residues within these clusters are highlyconserved evolutionarily. The lipid kinase activity of wild-type p110α or a "hot-spot"mutants (H1047R) (Fig. **2**) were measured. Expression of mutant p110αconferred more lipid kinase activity than expression of wild-typeprotein [387].These data suggested that mutant *PIK3CA* was likely to function asan oncogene in human cancers [387, 388]. This idea is consistent with previouslyreported alterations of members of the PI3K pathway, particularlyinactivation of the *PTEN* tumor suppressor [388]. This study found noevidence of *PIK3CA* gene amplification in 96 colorectal cancers, suggesting that amplification is not a common mechanism of activation in this tumor type [387]. A subsequent study analysed 340 genes coding for serine/threonine kinases in colorectal tumours [389]. A total of 23 changes, including a majority (20) of non-synonymous point mutations were identified [389]. The gene mutations affected eight different proteins, including PDK1 (3 mutations out of which two mutations affected the same residue in the kinase domain) and Akt2 (2 mutations) [389]. Eighteen of the 23 somatic mutations occurred at evolutionarily conserved residues in the genes encoding the serine/threonine kinases [389]. 

### Clinical Findings Related to *PIK3CA* Mutations

A large-scale mutational analysis of the helical and catalytic domains of *PIK3CA* was performed in brain tumors [390]. A total of 13 mutations of *PIK3CA* within these specific domains were identified in anaplastic oligodendrogliomas, anaplastic astrocytomas, glioblastoma multiforme, and medulloblastomas, whereas no mutations were identified in ependymomas or low-grade astrocytomas [390]. These observations implicated *PIK3CA* as an oncogene in a wider spectrum of adult and pediatric brain tumors and suggested that *PIK3CA* may be a useful diagnostic marker or a therapeutic target in these cancers [390].

Another group screened a large panel of primary human tumors for mutations in all coding exons of *PIK3CA* [391]. A strong proportion of primary breast cancers (40%) harbored mutations in *PIK3CA*, which were not associated with histologic subtype, estrogen receptor status, grade or presence of tumor in lymph nodes [391]. Among the primary epithelial ovarian cancers only 6.6% contain somatic mutations, but there was a clear histologic subtype bias in their distribution [391]. Only a minority (2.3%) of serous carcinomas had *PIK3CA* mutations compared with 20.0% of endometrioid and clear cell cancers. In contrast, *PIK3CA* gene amplification (>7-fold) was common among all histologic subtypes and was inversely associated with the presence of mutations [391]. Overall, *PIK3CA* mutation or gene amplification was detected in 30.5% of all ovarian cancers and 45% of the endometrioid and clear cell subtypes [391]. In advanced ovarian carcinomas, activating *PIK3CA* missense mutations were found in only about 4% of the cases [392]. Somatic missense mutations in *PIK3CA* were observed in 12% of ovarian carcinomas, and in 18% breast carcinomas in [393]. Another study reported *PIK3CA *somatic mutations in hepatocellular carcinomas (35.6%), breast carcinomas (26.9%), gastric carcinomas (6.5%), acute leukemias (1.1%) and non-small-cell lung cancers (1.3%) [394]. Some of the *PIK3CA* mutations were detected in the early lesions of breast cancer carcinoma, hepatocellular carcinoma, and gastric carcinomas, suggesting that *PIK3CA* mutation may occur independent of stage of the tumors [394]. *PIK3CA* mutations were identified in 26% of human breast tumor samples and cell lines at about equal frequency in tumor stages I to IV in another study [395]. A highly significant association between *PIK3CA* mutations and retention of PTEN protein expression was observed. In addition, *PIK3CA* mutations were associated with expression of estrogen and progesterone receptors, lymph node metastasis, and ErbB2 overexpression. The fact that *PIK3CA* mutations and *PTEN* loss were nearly mutually exclusive implied that deregulated PI3K signaling is critical for tumorigenesis in breast cancers and that loss of either *PIK3CA* or *PTEN *abrogates the selective pressure for targeting of the other gene. However, a subsequent study found that *PIK3CA* and *PTEN* mutations in breast cancer were not mutually exclusive and correlated with similar prognostic factors [396]. Intriguingly, *PIK3CA* mutations predicted for longer local recurrence-free survival [396]. In thyroid cancer, non-synonymous somatic mutations in *PIK3CA* were found in anaplastic thyroid carcinomas (23%) [397]. In endometrial carcinoma, *PIK3CA* mutations occurred at high frequency (36%) as did the coexistence of *PIK3CA/PTEN* mutations (26%) [398]. *PIK3CA* mutations were more common in tumors with *PTEN *mutations (46%) compared with those without *PTEN* mutations (24%) [398]. In head and neck squamous cell carcinoma, mutations in *PIK3CA* were reported in 11% of the cases analysed [399]. Three of the four mutations (H1047R, E542K, E545K) had been previously reported as hotspot mutations [399]. In pancreatic cancer a recent study described *PIK3CA* mutations in a fraction (11%) of the specimens analysed and some of the somatic mutations were novel [400]. 

### Validation of *PIK3CA* as an Oncogene

The oncogenic potential of three of the most commonly observed *PIK3CA* mutations (E542K, E545K, H1047R) was investigated in chicken embryo fibroblasts [401]. All three mutants induced oncogenic transformation with high efficiency [401]. The mutant-transformed cells displayed constitutive phosphorylation of Akt, of p70(S6K), and of the 4E-BP1 [401]. Rapamycin strongly suppressed cellular transformation induced by the PI3K mutants, suggesting that TOR and its downstream targets are essential components of the transformation process [401]. It was also shown that three prevalent mutants of p110α are oncogenic *in vivo* [402]. The mutants induced tumors in the chorioallantoic membrane of the chicken embryo and caused hemangiosarcomas in the animal [402]. These tumors were marked by increased angiogenesis and an activation of the Akt pathway [402]. The TOR inhibitor RAD001 blocked tumor growth induced by the H1047R p110α mutant [402]. In a subsequent study, all of eight *PIK3CA* mutations examined were shown increased p110α lipid kinase activity compared with the wild-type enzyme [403]. All the mutants strongly activated Akt and p70(S6K) and also induced morphologic changes, loss of contact inhibition, and anchorage-independent growth of NIH3T3 cells [403]. The hotspot mutations E542K, E545K, and H1047R, all had high enzymatic and transforming activities [403]. These results showed that almost all the colon cancer-associated *PIK3CA *mutations are functionally active so that they are likely to be involved in carcinogenesis [403]. In a recent report, a panel of rare *PIK3CA* mutants was evalulated in chicken fibroblast tansformation assays, which also revealed their oncogenic potential and mechanism of activation [404].

To evaluate the consequences of *PIK3CA* alterations, the two most common mutations were inactivated by gene targeting in colorectal cancer cells [405]. Biochemical analyses of these cells showed that mutant *PIK3CA* selectively regulated the phosphorylation of Akt and of forkhead transcription factors [405]. *PIK3CA *mutations had little effect on growth under standard conditions, but reduced cellular dependence on growth factors [405]. *PIK3CA* mutations resulted in attenuation of apoptosis and facilitated tumor invasion [405]. Importantly, treatment with LY294002 abrogated *PIK3CA* signaling and preferentially inhibited growth of *PIK3CA* mutant cells [405]. A recent study also showed that *PIK3CA *mutant colon cancer cell lines have increased metastatic potential in an orthotopic model [406]. Two *PIK3CA* mutants observed in breast cancer (E545K and H1047R) were also tested in the MCF-10A immortalized breast epithelial cell line [407]. Both variants displayed higher PI3K activity than wild-type p110α yet remained sensitive to pharmacologic PI3K inhibition [407]. In addition, expression of p110α mutants in mammary epithelial cells induced multiple phenotypic alterations characteristic of breast tumor cells, including anchorage-independent proliferation in soft agar, growth factor-independent proliferation, and protection from anoikis [407]. Expression of these mutant p110α isoforms also confered increased resistance to paclitaxel and induced abnormal mammary acinar morphogenesis in three-dimensional basement membrane cultures [407].

Another study compared the biochemical activity and transforming potential of mutant forms of p110α and p110β in a human mammary epithelial cell system [408]. The two most common tumor-derived alleles of p110α (H1047R and E545K) potently activated PI3K signaling [408]. Human mammary epithelial cells expressing these alleles grew efficiently in soft agar and as orthotopic tumors in nude mice [408]. A mutation in p110β homologous to the E545K allele of p110α was then constructed, but the resulting p110β mutant was only weakly activated and induced minimal soft-agar growth [408]. However, a gene fusion of p110β with a membrane anchor was highly active and transforming in both soft-agar and orthotopic nude mouse assays [408]. Work by another group investigated the oncogenic potential of all 4 catalytic class I PI3K isoforms [409]. At physiological levels of expression, the wild-type p110α isoform lacked oncogenic potential, but gain-of-function mutations and overexpression of p110α were correlated with oncogenicity [409]. The p110β, p110γ and p110δ isoforms induced transformation of cultured cells as wild-type proteins, which could be suppressed by rapamycin [409]. The p110δ isoform constitutively activated the Akt signaling pathway, but p110γ activated Akt only in the presence of serum [409]. The p110 isoforms also differed in their requirements for upstream (Ras) signaling [409]. 

## ONCOGENIC MUTANTS OF THE p85 ADAPTOR 

There exist several oncogenic mutants derived from the p85 adaptor. The first to be characterized was p65(PI3K), a mutant of the regulatory subunit of PI3K, which includes the initial 571 residues of the wild type p85α-protein linked to a region conserved in the Eph tyrosine kinase receptor family. It was demonstrated that this mutation, obtained from a transformed cell, induces the constitutive activation of PI3K and contributes to cellular transformation [410]. It was shown that transgenic mice expressing in T lymphocytes p65(PI3K) developed an infiltrating lymphoproliferative disorder and autoimmune renal disease with an increased number of T lymphocytes exhibiting a memory phenotype and reduced apoptosis [411]. An oncogenic fusion product of the p85β subunit of PI3K and HUMORF8, a putative deubiquitinating enzyme was also described in a chronic myeloproliferative disorder [412]. Single-strand conformational polymorphism/heteroduplex analysis revealed the presence of somatic mutations in the gene for the p85α regulatory subunit of PI3K (*PIK3R1*) in primary human colon and ovarian tumors and cancer cell lines [413]. All of the mutations lead to deletions in the inter-SH2 region of the molecule proximal to the Ser608 autoregulatory site [413]. Expression of a mutant protein with a 23 amino acid deletion leads to constitutive activation of PI3K providing the first direct evidence that p85α is a new oncogene involved in human tumorigenesis [413]. A C-terminal truncated form of p85 was described in a human lymphoma cell line (CO) with a T cell phenotype derived from a patient with Hodgkin's disease [414]. As a result of a frame-shift mutation at amino acid 636, p76 is lacking most of the C-terminal SH2 domain, but contains the inter-SH2 domain and is associated with an active form of PI3K [414]. A PI3K-dependent constitutive activation of Akt was detected in CO cells, which was only partially reduced after serum starvation [414]. Treatment of CO cells with the PI3K inhibitor wortmannin resulted in a concentration-dependent inhibition of cell proliferation associated with an increased number of apoptotic cells [414].

## PI3K INHIBITORS AS ANTI-TUMOR AGENTS

Wortmannin and LY294002 were evaluated as potential anti-tumor agents in numerous studies starting in the mid-nineties [415] and their ability to sensitizes tumor cells to chemotherapy and radiotherapy has been well documented [416]. The growth of Philadelphia chromosome (Ph)-positive leukemic cells was inhibited by wortmannin [417]. PI3K inhibition sensitized human tumor cells to the effects of radiation [418]. Since wortmannin decreases the activities of both the ATM protein and DNA-PK, it was suggested that it might be of use as a sensitizing agent for radiotherapy and chemotherapy [121, 419]. The double-strand break signaling/repair proteins ATM, ATR, and DNA-dependent protein kinase catalytic subunit (DNA-PK) are attractive targets to confer enhanced radio and chemosensitivity to tumor cells. Small interfering RNA (siRNA) targeting ATM and DNA-PK gave rise to a significant dose-reduction factor compared at the clinically relevant radiation doses, which was greater than the radiosensitivity achieved using wortmannin or LY294002 [420]. A similar increased sensitivity to the alkylating agent methyl methanesulfonate (MMS) was also observed for siRNA-mediated ATR silencing [420].

In Ewing's sarcoma family of tumors (ESFTs) the PI3K inhibitor wortmannin and LY294002 were evaluated in combination with chemotherapy [421]. Doxorubicin treatment reduced cell number and enhanced apoptosis in PI3K inhibited cells compared with noninhibited cells [421]. In leukemia, the PI3K inhibitors LY294002 and wortmannin induced a significant increase in apoptosis in combination with cytotoxic drugs [422]. The PI3K/Akt pathway was shown to play a significant role in mediating drug resistance in human pancreatic cancer cells [423]. PI3K inhibitors were proposed to have therapeutic potential when combined with the chemotherapy agent gemcitabine in the treatment of pancreatic cancers [423]. 

Another report showed that cisplatin-induced DNA damage causes the phosphorylation of BAD Ser-136 *via* a PI3K/Akt cascade and that inhibition of either of these cascades sensitizes ovarian cancer cells to cisplatin [424]. Further work suggested that paclitaxel induces the phosphorylation of BAD Ser-112 *via* the Erk cascade, and the phosphorylation of both BAD Ser-136 and Raf-1 Ser-259 *via* the PI3K/Akt cascade, and that inhibition of either of these cascades sensitizes ovarian cancer cells to paclitaxel [425]. These observations were further developed in an *in vivo* model in [426]. LY294002 in combination with carboplatin was more effective in inhibiting ovarian cancer cell xenograft growth than either agent alone [426]. 

In breast cancer cell lines, Akt activity was constitutive and was associated with either *PTEN* mutations or ErbB2 over-expression [427]. When combined with therapies commonly used in breast cancer treatment, LY294002 potentiated apoptosis caused by doxorubicin, trastuzumab, paclitaxel, or etoposide [427]. Potentiation of apoptosis by LY294002 correlated with induction of Akt by doxorubicin or trastuzumab alone that occurred before the onset of apoptosis [427]. To determine a causal relationship between the activity of Akt and radioresistance in human breast cancer cells, MCF7 cells, transfected with constitutively active H-Ras (RadG12V) or constitutively active Akt, were chosen for analysis in [428]. The results from this study indicated that the expression of constitutively active Ras and the expression of constitutively active Akt each increased cellular resistance to radiation [428]. Inhibition of PI3K with LY294002 reverted the constitutively active Ras-mediated radioresistance, but not the constitutively active Akt-mediated radioresistance [428]. These data suggested that Akt may be a potential target for enhancing the response to radiotherapy in patients with breast cancer [428]. Another study showed the ErbB2/P13K/Akt pathway plays a role in mediating multi-drug resistance in human breast cancer cells [429]. It was found that cell lines that express both ErbB2 and ErbB3 appear to have a higher phosphorylation level of Akt [429]. Transfection of ErbB2 in MCF7 breast cancer cells that express ErbB3 caused a PI3K-dependent activation of Akt, and was associated with an increased resistance of the cells to multiple chemotherapeutic agents (paclitaxel, doxorubicin, 5-fluorouracil, etoposide, and camptothecin) [429]. Selective inhibition of PI3K or Akt activity with their respective dominant-negative expression vectors sensitized the cells to the induction of apoptosis by the chemotherapeutic agents [429].

Another study reported constitutive Akt phosphorylation (on Ser473) consistent with pathway activation in human pancreatic carcinoma cell lines *in vitro *[430]. Exposure of the cells to wortmannin and LY294002 resulted in a dose-dependent induction of apoptosis in six of seven of the cell lines that displayed constitutive Akt phosphorylation but not in either of the cell lines that did not [430]. Exposure of orthotopic pancreatic tumors to LY294002 resulted in dose-dependent inhibition of tumor growth, and decreased peritoneal and liver metastases, effects that were associated with an inhibition of Akt phosphorylation and increased apoptosis [430]. Furthermore, a suboptimal dose of LY294002 produced additive inhibition of tumor growth when combined with a suboptimal dose of gemcitabine [430]. A further report demonstrated that PI3K/Akt signaling promotes SCLC growth, survival, and chemotherapy resistance [431]. A panel of SCLC cell lines was very sensitive to LY294002 and the inhibitor potentiated the effect of low concentrations of etoposide in inhibiting growth and inducing apoptosis [431]. In AML, a HL-60 cell clone was isolated which was highly resistant to several drugs inducing apoptosis and to the differentiating chemical all-trans-retinoic acid (ATRA) [432]. The resistant clone displayed an activated PI3K/Akt pathway, with levels of PIP_3_ higher than the parental cells and increased levels of phosphorylated Akt1 [432]. Treatment of the resistant cell clone with wortmannin or LY294002 reversed resistance to drugs [432]. Resistant cells over-expressing either dominant negative PI3K or dominant negative Akt1 became sensitive to drugs and ATRA [432]. A subsequent study investigated whether a HL-60 human leukemia cell clone (named AR) with constitutively active Akt1 was resistant to TRAIL [433]. Parental HL-60 cells were very sensitive to TRAIL and died by apoptosis, while AR cells were resistant to TRAIL. LY294002 and wortmannin restored TRAIL sensitivity of AR cells [433]. Another study investigated the molecular mechanisms by which drug resistance develops in human prostate cancer cells with a subline selected for resistance to camptothecin [434]. Expression differences were found in apoptosis-related genes in the direction expected on the basis of the apoptosis-resistance for BAD, caspase-6, and genes that signal *via* the Akt pathway [434]. Exposure of the cells to wortmannin provided functional support for involvement of the Akt pathway [434].

Another study demonstrated synergistic augmentation of the cytotoxicity by LY294002 occurs specifically with antimicrotubule agents, at least partially through an increase in caspase 3-dependent apoptosis, and suggested that inhibitors of the PI3K/Akt pathway in combination with antimicrotubule agents may induce cell death effectively and be a potent modality to treat patients with malignant gliomas [435].

Cells transformed by oncogenic Ras, display a radioresistant phenotype in response to ionizing radiation (IR). The PI3K inhibitor LY294002 radiosensitized cells bearing mutant Ras, but the survival of cells with wild-type Ras was not affected [436]. In another study, the mechanisms by which Ras mediates radioresistance in epithelial cells were assessed [437]. The importance of three major survival pathways that can be activated by Ras (PI3K/Akt, NF-κB, and Raf/MEK/Erk) were investigated as necessary or sufficient for Ras-mediated radioresistance in matched pairs of epithelial cells expressing oncogenic Ras or empty vector [437]. Inhibiting PI3K with LY294002 sensitized cells expressing oncogenic Ras to IR, indicating that PI3K is necessary for Ras-mediated radioresistance [437]. Another study suggested that a combination of the PI3K inhibitor LY294002 and conventional chemotherapy (paclitaxel) may provide an effective approach to inhibiting tumor growth and ascites production in ovarian cancer with acceptable side effects [438]. LY294002 demonstrated a growth-inhibitory and apoptosis-inducing effect in colon cancer cell lines, with decreased expression of phosphorylated Akt [439]. In experiments using mouse xenografts, it was found that LY294002 administration *in vivo* also resulted in suppression of tumor growth and induction of apoptosis [439]. Resistance to conventional adjuvant therapies (chemotherapy and radiation) has been well documented in malignant gliomas. *In vitro*, an actual antagonistic effect between sequential administration of radiation and 1,3-bis(2-chloroethyl)-1-nitrosourea (BCNU) chemotherapy in primary human glioblastoma cell lines was observed, which was abrogated upon inhibition of EGFR with the a tyrosine kinase inhibitor [440]. It was found that BCNU inhibited radiation-induced apoptosis through EGFR-mediated activation of PI3K/Akt *via* Ras [440]. 

Several reports have suggested an involvement of the Ras/PI3K/Akt signalling pathways in controlling multidrug resistance. It was found found cotreatment of drug-resistant colon cancer cells with the topoisomerase inhibitor doxorubicin and LY294002 resulted in massive apoptosis, suggesting that the PI3K pathway controls cell survival and drug resistance in these cells [441]. LY294002 inhibited drug export in a competitive manner and the efficacy of drug efflux inhibition by LY294002 was similar to that achieved by MRP1 inhibitors [441].

Further work provided the first evidence that the systemic administration of a PI3K inhibitor LY294002 has antitumor and antiangiogenic activity *in vivo* [442]. It was shown that PTEN reconstitution diminished phosphorylation of Akt, induced the transactivation of p53 and increased the expression of p53 target genes in glioma cells [442]. PTEN and LY294002 induced p53 activity in human brain endothelial cells, suggesting that PTEN and PI3K pathways can suppress the progression of cancer through direct actions on tumor and endothelial cells [442]. LY294002 inhibited glioma tumor growth *in vivo*, induced tumor regression, decreased the incidence of brain tumors, and blocked the tumor-induced angiogenic response *in vivo* [442]. These data provided evidence that both PTEN and PI3K inhibitors regulate p53 function and display *in vivo* anti-angiogenic and anti-tumor activity [442]. These results provided evidence that the two tumor suppressor genes, PTEN and p53, act together to block tumor progression *in vivo*. LY294002 inhibited ascites formation in a mouse model of human ovarian cancer by inhibiting tumor and peritoneal neovascularization as well as vascular permeability [443]. LY294002 also directly inhibited VEGF protein expression and release from ovarian carcinoma, suggesting that the inhibitor blocks the VEGF signaling pathway involved in angiogenesis and vascular permeability [443].

Topical treatment with inhibitors of the PI3K/Akt and Raf/mitogen-activated protein kinase kinase/extracellular signal-regulated kinase pathways inhibited the growth of TPras transgenic melanomas in severe combined immunodeficient mice, blocked invasive behavior, and reduced angiogenesis [444]. The inhibitor LY294002 effectively reduced melanoma cell growth both *in vitro* and *in vivo* [444]. Both LY294002 and U0126, a MEK 1/2 inhibitor, reduced invasion, which correlated with reduction of the metalloproteinase matrix metalloproteinase 2 [444]. Tumor angiogenesis was disrupted through inhibition of VEGF production from the tumor cells and antiangiogenic effects on endothelial cells [444].

STI571 (Gleevec), an anti-leukemia drug targeting BCR/ABL kinase can induce remissions of the Ph(1)-positive leukemias (CML). The anti- Ph(1)-leukemia effect of the combination of BCR/ABL kinase inhibitor STI571 and PI3K inhibitors wortmannin or LY294002 was tested in [445]. It was shown that STI571 and wortmannin exerted a synergistic effect against the Ph(1)-positive cell lines, but did not affect the growth of Ph(1)-negative cell line [445]. Moreover, the combinations of STI571 and wortmannin or STI571 and LY294002 were effective in the inhibition of clonogenic growth of CML-chronic phase and CML-blast crisis patient cells, while sparing normal bone marrow cells [445].

In addition, novel PI3K inhibitors have been described, that may be more potent than LY294002 or wortmannin. For example, ZSTK474 has recently been described as a new PI3K inhibitor with strong antitumor activity against human cancer xenografts without toxic effects in critical organs [446]. 

The potential of deguelin, a natural plant product, as a lung cancer chemopreventive agent was investigated in [447]. Deguelin treatment inhibited the growth of and induced apoptosis of premalignant and malignant human bronchial epithelial (HBE) cells, but had minimal effects on normal HBE cells [447]. Levels of phosphorylated Akt (pAkt) were higher in premalignant HBE cells than in normal HBE cells [447]. In premalignant HBE cells, deguelin inhibited PI3K activity and reduced phosphorylated Akt levels and activity. A constitutively active Akt in premalignant and malignant HBE cells blocked deguelin-induced growth arrest and apoptosis [447]. Because both premalignant and malignant HBE cells are more sensitive to deguelin than normal HBE cells, deguelin may have potential as both a chemopreventive agent for early stages of lung carcinogenesis and a therapeutic agent against lung cancer [447]. In AML, a further study showed that deguelin induced cell cycle arrest and increased apoptotic cell death [448]. Deguelin also downregulated Akt phosphorylation of leukaemia cells and markedly increased sensitivity of U937 cells to etoposide or cytarabine [448].

## ISOFORM-SPECIFIC PI3K INHIBITORS

There is extensive evidence from the molecular and genomic analysis of human cancers presented above that the PI3K/Akt pathway is deregulated in malignant progression [449]. Furthermore, the causal involvement of PI3K is supported by gene-knockout mouse models. Prototype inhibitors such wortmannin and LY294002 have show evidence of anticancer activity *in vitro* and *in vivo* animal models. Therefore, the recent development of isoform-selective inhibitors shows considerable promise for cancer treatment [450-452].

To determine the contribution of the hemopoietic cell-restricted p110δ in neutrophil chemotaxis, a potent and selective p110δ inhibitor, IC87114 was developed [453, 454]. IC87114 inhibited polarized morphology of neutrophils, fMLP-stimulated PIP_3_ production and chemotaxis [453]. Isoform-selective PI3K p110β inhibitors were developed, which prevented formation of stable integrin α(IIb)β(3) adhesion contacts in platelets, leading to defective platelet thrombus formation [455]. A new generation of isoform-selective class I PI3K inhibitors was used to show that reactive oxygen species production in neurophils is regulated by temporal control of class I PI3K activity [456]. Further studies described the identification and development of specific, selective and orally active small-molecule inhibitors of p110γ with activity in mouse models of rheumatoid arthritis [457]. A chemically diverse panel of PI3K inhibitors was synthesized and evaluated in a recent study [458]. It was found that p110α is the primary insulin-responsive PI3K in cultured cells, whereas p110β was dispensable, but set a phenotypic threshold for p110α activity. Compounds targeting p110α impaired the acute effects of insulin treatment *in vivo* [458]. Intriguingly, these results corroborated the findings from a study using a knock-in mouse with a catalytically-inactive p110α [459].

In a first study in human cancer, a large panel of tissues and cell lines were screened for expression of class I_A_ PI3Ks, which documented a ubiquitous expression of the p110α and p110β isoforms but a variable and more restricted tissue distribution of the p110δ isoform [460]. Originally found in leukocytes, p110δ was also detected in some nonhematopoietic cell types especially those of breast or melanocytic origin, both in the untransformed and transformed state [460]. Isoform-specific neutralization of PI3K isoforms in breast cancer cell lines, by PI3K antibody microinjection or a p110δ-selective pharmacological inhibitor, demonstrated that p110δ is the most important class I_A_ PI3K in the regulation of epidermal growth factor-driven motility *in vitro*, controlling the directionality and, to a lesser extent, the speed of migration [460]. In contrast, p110β was required for the direction but not the speed of migration, whereas p110α did not impact on either of these parameters [460]. These results showed a nonredundant function of PI3K isoforms downstream of the epidermal growth factor receptor and indicate that the presence of p110δ may confer breast cancer cells with selective migratory capacities [460].

The p110δ isoform was shown to be expressed in endothelial cells [461]. The p110δ specific inhibitor IC486068 abrogated radiation-induced phosphorylation of Akt. IC486068 enhanced radiation-induced apoptosis in endothelial cells and reduced cell migration and tubule formation of endothelial cells in Matrigel following irradiation [461]. IC486068 enhanced radiation-induced endothelial cytotoxicity, resulting in tumor vascular destruction and tumor control when combined radiotherapy in murine tumor models [461]. These findings suggest that p110δ is a therapeutic target to enhance radiation-induced tumor control [461].

It was shown that the p110δ isoform is consistently expressed at a high level in blast cells from AML, in contrast to the other class I isoforms, the expression of which was very variable among patients [462]. The p110δ-selective inhibitor IC87114 suppressed both constitutive and Flt-3-stimulated Akt activation in blasts to the same extent as LY294002, an inhibitor of all PI3K isoforms. Moreover, IC87114 inhibited AML cell proliferation without affecting the proliferation of normal hematopoietic progenitor cells [462]. Thus, p110δ may represent a potential therapeutic target in AML. In a subsequent study, the combination of IC87114 and etoposide/VP16 was shown to be synergistic in reducing cell viability in AML, and was associated with a reduction in constitutive NF-κB activity [463]. 

Several other isoform-specific PI3K inhibitors have been developed in the past years. Biologically stable semisynthetic viridins were developed as inhibitors of PI3K in [464]. The most active compound was termed PX-866 and is a PI3K inhibitor with selectivity for p110α [464]. Initial results showed that PX-866 is a biologically stable broad-spectrum PI3K inhibitor with good pharmacokinetics that causes prolonged inhibition of PI3K signaling in human tumor xenografts [464]. PX-866 exhibited single agent *in vivo* antitumor activity and increased the antitumor effects of cisplatin and radiation treatment [464]. In a subsequent study, PX-866 was shown to potentiate the antitumor activity of gefitinib against NSCLC xenografts *in vivo* [465].

In a recent study, one single agent (PI-103) induced proliferative arrest in glioma cells, despite the ability of many other compounds under study to block PI3K signaling through its downstream effector, Akt [466]. The unique cellular activity of PI-103 could be attributed to its ability to inhibit both p110α and mTOR [466]. PI-103 showed significant activity in tumors *in vivo* with no observable toxicity [466]. These data demonstrated an emergent efficacy due to combinatorial inhibition of mTOR and p110α in malignant glioma [466].

## Akt INHIBITORS

A small molecule Akt pathway inhibitor, Akt/protein kinase B signaling inhibitor-2 (API-2) suppressed the kinase activity and phosphorylation level of Akt [467]. The inhibition of Akt kinase resulted in suppression of cell growth and induction of apoptosis in human cancer cells that harbor constitutively activated Akt due to overexpression of Akt or other genetic alterations such as *PTEN* mutation [467]. API-2 was highly selective for Akt and did not inhibit PI3K activation or other kinases [467]. Furthermore, API-2 potently inhibited tumor growth in nude mice of human cancer cells in which Akt is aberrantly expressed/activated but not of those cancer cells in which it is not [467]. 

Another report investigated the effects of small molecule inhibitors of Akt, which target the Akt PH domain and have isoform-specific activity [468]. In multiple cellular backgrounds, maximal induction of caspase-3 activity, an indicator of apoptosis, was achieved when both Akt1 and Akt2 were inhibited [468]. It was also shown that in different tumor cell backgrounds inhibition of mTOR was less effective in inducing caspase-3 activity than inhibition of Akt1 and Akt2 [468].

A study examined whether Akt is required and sufficient to mediate brain-derived neurotrophic factor (BDNF)/TrkB protection of neuroblastoma cells from chemotherapy [469]. Pharmacologic inhibition of Akt, with PIA6, a phosphatidylinositol ether lipid analogue, blocks BDNF-induced phosphorylation of Akt and the downstream target of Akt [469]. PIA6 sensitizes neuroblastoma cells to chemotherapy and attenuates BDNF protection of neuroblastoma cells from chemotherapy-induced cell death [469].

KP372-1, another Akt inhibitor, was evaluated in thyroid cancer cells [470]. Thyroid cancer consistently expresses phosphorylated Akt. KP372-1 blocked signalling downstream of Akt in thyroid tumour cells, leading to inhibition of cell proliferation and increased apoptosis [470]. In another study, KP-372-1 and KP-372-2 effectively inhibited the PI3K/Akt signaling cascade in glioblastoma [471]. Furthermore, the treatment of glioma cells with these inhibitors resulted in the induction of apoptosis [471].

## RAPAMYCIN AND RAPAMYCIN ANALOGS

The mammalian target of rapamycin (mTOR) has emerged as an important therapeutic target for cancer [472-474]. Rapamycin and its derivatives that specifically inhibit mTOR are now being actively evaluated in clinical trials [472, 474]. However, many cancer cells are resistant to rapamycin and its derivatives. The mechanism of this resistance remains a subject of major therapeutic significance [475]. We will not discuss the potential of rapamycin and its derivatives in great detail here, in view of the many excellent reviews already published on the topic.

It was reported that the inhibition of mTOR by rapamycin triggers the activation of two survival signaling pathways that may contribute to drug resistance. Treatment of human lung cancer cells with rapamycin suppressed the phosphorylation of p70(S6K) and 4E-BP1, indicating an inhibition of mTOR signaling [476]. Paradoxically, rapamycin also concurrently increased the phosphorylation of both Akt and eIF4E [476]. The rapamycin-induced phosphorylation of Akt and eIF4E was suppressed by LY294002, suggesting the requirement of PI3K in this process [476]. The activated Akt and eIF4E appeared to attenuate the growth-inhibitory effects of rapamycin, serving as a negative feedback mechanism. In support of this model, rapamycin combined with LY294002 exhibited enhanced inhibitory effects on the growth and colony formation of cancer cells [476]. Further work suggested that feedback down-regulation of receptor tyrosine kinase signaling is a frequent event in tumor cells with constitutive mTOR activation [477]. Reversal of this feedback loop by rapamycin may attenuate its therapeutic effects, whereas combination therapy that ablates mTOR function and prevents Akt activation may have improved antitumor activity [477]. A recent report showed that that rapamycin mediates Akt activation through an IGF-IR-dependent mechanism [478]. Thus, it was concluded that combinatorial targeting of mTOR and the IGF-IR may be a promising strategy to enhance mTOR-targeted anticancer therapy [478].

## CONCLUSIONS AND PERSPECTIVES

Almost twenty years after the discovery of PI3K, this family of enzymes has become the focus of intensive research aimed at developing new drugs for a wide variety of diseases, including cancer, inflammation and allergy. The discovery of the many genetic alterations affecting the PI3K/PTEN/Akt/mTOR pathway in human cancer has clearly placed some of the enzymes involved, including p110α, mTOR and Akt, on the top list of druggable targets in the field on oncology research. Moreover, the recognition that some of the molecular alterations, such as increased Akt phosphorylation or *PTEN* mutations correlate with clinical parameters, such as outcome, in cancer patients will possibly result in the development of new diagnostic protocols in specific types of cancer. What are the challenges ahead? The generic PI3K inhibitors LY294002 and wortmannin have greatly facilitated the deciphering of the biological responses and signaling pathways controlled by PI3K and have even proved to be successful in a variety of pre-clinical models of human cancer. The new generations of pharmacological inhibitors with increased specificity for one or a restricted number of PI3K isoforms and related kinases are entering clinical trials. It has been demonstrated that combining these agents with receptor tyrosine kinase inhibitors, which have already been evaluated in clinical trials, can produce synergistic effects in stopping tumor growth in a variety of pre-clinical models. One can thus reasonably hope that these drug combinations will result in improved survival benefits for cancer patients. However, there remain many open questions related to the mechanisms governing sensitivity or resistance of tumors to these targeted agents. The best examples of such open questions are rapamycin and its analogs. There are now several reports describing potential novel mechanisms of resistance to these agents and there may be even more to be discovered in the future. A central question in the field of isoform-specific PI3K inhibitors is the question of specificity. The class I_A_ PI3K catalytic isoforms have a high degree of sequence homology in their catalytic domains and demonstrating the specificity of the cellular effects of these agents remains problematic, since there are no specific readouts that discriminate between these three enzymes. Moreover, assessing the exact expression pattern of all enzymes of the PI3K family in a given tumor sample remains problematic, in view of the lack of availability of isoform-specific antibodies which can be used in immunohistochemistry. Such information is in our opinion of great importance, since there exist, in addition to the well-studied class I PI3K isoforms, three class II enzymes, with still poorly understood cellular functions, at least in human cancer. In addition, the class III PI3K Vps34p should also be taken into account when designing future therapies based on targeting PI3K isoforms, in view of its roles in autophagy and intracellular trafficking events.

## Figures and Tables

**Table 1.** Genetic Alterations in the PI3K/Akt Pathway in Cancer

Pathway Component	Type of Alteration	Tumor Lineage	References
**PTEN**	Loss-of-function by somatic mutation	Brain, prostate, endometrium	[ 479-481]
	Germline mutation (in 80% of Cowden Disease)	Cowden disease: Increased risk for breast, thyroid, genitourinary and endometrial cancer	[ 482, 483]
	Transcriptional down-regulation (e.g., promoter methylation)	Melanoma, breast, colon	[484-486]
	Loss of heterozygosity	Prostate, melanoma, thyroid, breast, pancreas, ovary, brain, bladder, endometrium, cervix, head and neck, kidney, lung	Reviewed in [487]
**p110α**	Gain-of-function by somatic mutation	Colon, breast, brain, ovary	[387, 390]
	Amplification	Ovary, gastric, lung, cervix	[382, 391, 488, 489]
**p85**	Gain-of-function by somatic mutation	Brain, colon, ovary	[413, 490]
**AKT1**	Gain-of-function by somatic mutation	Breast, colorectal, ovary	[491]
**AKT2**	Amplification	Ovary, lymphoma, pancreas	[492-495]
	Mutation	Colorectal	[389]
**PDK1**	Mutation	Colorectal	[389]
**TSC 1/2**	Loss-of-function by mutation (occasionally with concomitant loss of heterozygosity for the wild type allele)	Tuberous sclerosis (hamartomas of the skin, brain and kidney; rare progression to malignancy)	[224]
**TSC1**	Mutation	Bladder	[496]
